# A phase 2 pilot study of umbilical cord blood infusion as an adjuvant consolidation therapy in elderly patients with acute myeloid leukemia

**DOI:** 10.1038/s41392-024-02065-y

**Published:** 2024-12-20

**Authors:** Jinzeng Wang, Xiaoyang Li, Ping Liu, Yao Dai, Hongming Zhu, Yunxiang Zhang, Min Wu, Yunying Yao, Mingzhu Liu, Shuting Yu, Fangying Jiang, Shuai Wang, Haoran Mu, Bo Jiao, Hua Yan, Wen Wu, Yang Shen, Junming Li, Shengyue Wang, Ruibao Ren

**Affiliations:** 1 Shanghai Institute of Hematology, State Key Laboratory for Medical Genomics, National Research Center for Translational Medicine at Shanghai, Ruijin Hospital, Shanghai Jiao Tong University School of Medicine, Shanghai, 200025 China; 2 Shanghai Jiao Tong University School of Medicine, Shanghai, 200025 China; 3 Department of General Practice, Ruijin Hospital, Shanghai Jiao Tong University School of Medicine, Shanghai, 200025 China; 4 Collaborative Innovation Center of Hematology, Ruijin Hospital, Shanghai Jiao Tong University School of Medicine, Shanghai, 200025 China; 5 International Center for Aging and Cancer, Hainan Medical University, Haikou, Hainan Province 571199 China

**Keywords:** Clinical trials, Haematological cancer, Haematological cancer

## Abstract

Acute myeloid leukemia (AML) is an aging-related malignancy, with patients aged ≥60 years old facing significantly poorer prognosis. Umbilical cord blood (UCB) has emerged as a promising source with effective anti-aging roles. Here, we conducted a prospective, phase 2, single-arm trial of UCB infusion as an adjuvant consolidation therapy in elderly AML patients (ChiCTR-OPC-15006492). A total of 51 patients were enrolled (median age 66 years; range, 60–75) and received two cycles of consolidation chemotherapy combined with UCB infusion. At a median follow-up of 27.3 months (range, 9.3–100), the median overall survival (OS) was not yet reached and the median event-free survival (EFS) was 72.2 months (range, 5.4–100). The 2-year OS and EFS rates were 76.9% and 62.8%, respectively. No acute graft-versus-host disease (aGVHD) or toxicity-related death occurred in any patient. The median times to platelet and neutrophil recovery were 11.5 days (range, 6–17) and 12.2 days (range, 0–21), respectively. Single-cell RNA sequencing (scRNA-seq) identified enhanced anti-tumor and anti-aging properties of UCB, manifested through activation of immune responses and telomere synthesis/maintenance. These findings suggest that UCB infusion is an effective and safe post-remission adjuvant therapy for elderly AML patients. This study provides evidence that anti-aging therapy may serve as a new and promising dimension in combined cancer treatment.

## Introduction

Acute myeloid leukemia (AML) is an aging-related heterogeneous malignancy, with abnormal proliferation of poorly differentiated myeloid cells.^1,2^ The global age-standardized incidence rate was about 1.73/100,000, while American and China had the highest incidence cases of AML.^3^ Treatments of AML consist of initial induction therapy and post-remission therapy. The backbone of induction therapy is 7 days of cytarabine with anthracycline (idarubicin, daunorubicin) for 3 days, known as “7 + 3” regimen.^4,5^ This results in a complete remission (CR) rate of 60–85% in patients less than 60 years of age, compared to only 40–60% in elderly patients (age ≥60 years old).^2,5^ Despite a high initial CR rate, most patients eventually succumb to relapsed AML, which is the most common reason of treatment failure in the management of AML.^6^ Older patients face a much higher relapse rate of 80–90% compared to less than 50% in younger patients with AML, resulting in highly unsatisfactory outcomes.^7,8^

Appropriate post-remission therapy is critical once CR is achieved after induction therapy. Common post-remission treatment strategies include additional cytotoxic chemotherapies (such as intermediate or high dose of cytarabine), or allogenic hematopoietic stem cell transplantation (allo-HSCT).^1^ Of those, allo-HSCT is the potential option for cure in patients with AML. For patients with an estimated relapse risk exceeding 35%, allo-HSCT is advised as the preferred consolidation therapy.^9^ However, allo-HSCT is related to increased risks of non-relapse mortality (NRM), due to complications such as chronic or acute graft-versus-host disease (GVHD), secondary malignancy, or infection caused by immunosuppression. Decision-making of whether to perform allo-HSCT is complicated, depending on the risks-benefits ratio of AML patients. Most patients with AML aged ≥ 60 years cannot proceed to allo-HSCT, due to multiple reasons including lack of donor, personal choice and biological factors. There remains an unmet medical need in elderly patients with AML, who are unable or unwilling to undergo allo-HSCT. It is imperative to develop novel and effective post-remission therapeutic regimens for these patients.

Umbilical cord blood (UCB) has been shown to have impressive anti-aging effects, including potentials to slow age-related degradation of cognitive functions and rejuvenate senescing phenotypes.^10,11^ In addition, usages of UCB have been increasingly studied for novel indications in malignancies,^12^ hemoglobinopathies,^13^ primary immune deficiency,^14^ regenerative medicine,^15^ and other genetic metabolic disorders.^16–18^ Banking of UCB has become a popular option and continues to rise worldwide.^19^ Important advances have been made in identifying UCB as a viable alternative for allo-HSCT, especially for patients who lack appropriate matched donors.^5,20^ UCB has distinct practical advantages, including ease of collection, rapid availability, more tolerant of Human Leukocyte Antigen (HLA) mismatches, and lower risks of GVHD.^21,22^ Given its accessibility, UCB can be available for most of the patients with AML in need.

In this study, we conducted a prospective, open label, phase 2, single-arm trial to investigate the efficacy and safety of UCB infusion as an adjuvant consolidation regimen in elderly AML patients. The primary endpoint assessed was overall survival (OS), with secondary endpoints including event-free survival (EFS), treatment-related toxicities, and median times to platelet and neutrophil recovery. Single-cell RNA sequencing (scRNA-seq) of matched samples collected before and after UCB infusion was carried out as an exploratory endpoint, providing insights into the cellular mechanisms underlying the therapeutic effects.

## Results

### Baseline characteristics of patients and treatment disposition

**Fig. 1:**
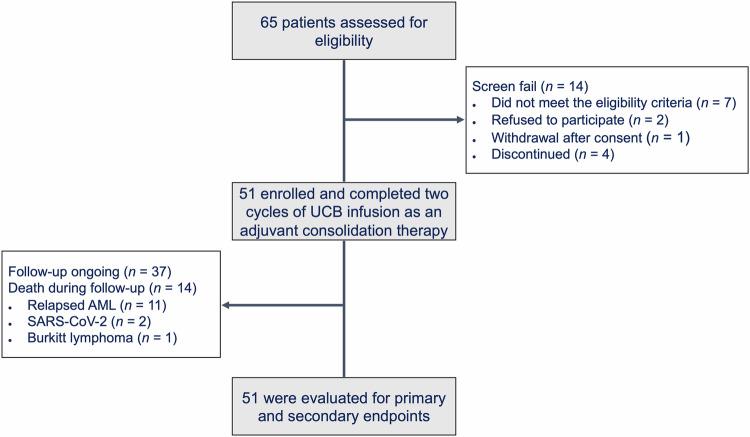
CONSORT flow diagram. Study diagram depicts the disposition of patients, including screening, consolidation therapy and follow-up

Between 12 January 2015 and 12 February 2022, a total of 65 elderly patients diagnosed newly with de novo AML were assessed for eligibility and 51 were enrolled in the trial of UCB infusion as an adjuvant consolidation therapy at Ruijin Hospital affiliated to Shanghai Jiao Tong University School of Medicine (Fig. 1). The full list of inclusion/exclusion criteria is presented in “Methods”. Baseline characteristics and treatment disposition of the participants are shown in Table 1. Of the 51 patients, 30 (58.8%) were female, and 21 (41.2%) were male, with a median age of 66 years (range, 60–75 years).

**Table 1:** Baseline characteristics of patients and treatment disposition

Variable	*n* = 51
Age, median (range), years	66 (60–75)
Gender, *n* (%)	
Male	21 (41.2)
Female	30 (58.8)
FAB subtypes, *n* (%)	
M0-M1	4 (7.8)
M2	24 (47.1)
M4	12 (23.5)
M5	8 (15.7)
M6-M7	3 (5.9)
ECOG, *n* (%)	
0–1	47 (92.2)
2	4 (7.8)
HCT-CI score, *n* (%)	
0	26 (51.0)
1/2	21 (41.2)
≥3	4 (7.8)
ELN 2022 risk classification, *n* (%)	
Favorable	15 (29.4)
Intermediate	26 (51.0)
Adverse	10 (19.6)
Karyotype, *n* (%)	
Normal karyotype	35 (68.6)
Complex karyotype	5 (9.8)
t(8;21)(q22;q22)	4 (7.8)
–9/del(9q)	2 (3.9)
+8	2 (3.9)
t(11;19)(q23;p13)	1 (2.0)
t(6;11)(q27;q23)	1 (2.0)
inv(16)(p13.1q22)/t(16;16)(p13.1;q22)	1 (2.0)
Selected molecular mutation, *n* (%)	
*NPM1*	12 (23.5)
*CEBPA*	12 (23.5)
*DNMT3A*	7 (13.7)
*TET2*	6 (11.8)
*IDH1*	6 (11.8)
*FLT3*-ITD	6 (11.8)
*IDH2*	3 (5.9)
*WT1*	3 (5.9)
*RUNX1*	3 (5.9)
*ASXL1*	2 (3.9)
*TP53*	1 (2.0)
Induction therapies, *n* (%)	
IA	43 (84.3)
VA	7 (13.7)
DHAG	1 (2.0)
MRD after induction therapies, *n* (%)	
Negative	38 (74.5)
Positive	13 (25.5)

*FAB* French-American-British, *ECOG* Eastern Cooperative Oncology Group, *HCT-CI* hematopoietic cell transplantation comorbidity index, *ELN* European Leukemia Net, *IA* idarubicin and cytarabine therapy, *VA* venetoclax plus azacytidine therapy, *DHAG* decitabine combined with homoharringtonine, cytarabine and granulocyte colony stimulating factor (G-CSF) therapy, *MRD* measurable residual disease

Patients were diagnosed according to World Health Organization (WHO) 2022 and French-American-British (FAB) criteria.^23,24^ Distribution of FAB subtypes included M0-M1 in 4 (7.8%), M2 in 24 (47.1%), M4 in 12 (23.5%), M5 in 8 (15.7%), and M6-M7 in 3 (5.9%) patients. The Eastern Cooperative Oncology Group (ECOG) performance status of 0–1, and 2 were found in 47 (92.2%) and 4 (7.8%) patients, respectively. Prognostic risk groups were classified based on molecular and cytogenetic abnormalities in the European LeukemiaNet (ELN) 2022 recommendations.^9^ Fifteen (29.4%) patients had favorable prognosis, 26 (51.0%) patients displayed intermediate prognosis, and 10 (19.6%) patients exhibited adverse prognosis. Fluorescence in situ hybridization (FISH) analysis of pretreatment bone marrow samples detected cytogenetic abnormalities of complex karyotype in 5 (9.8%) patients, based on the International System of Human Cytogenetic Nomenclature.^25^ The most common mutations in the patients were *NPM1* (23.5%), *CEBPA* (23.5%) and *DNMT3A* (13.7%). Prior induction therapies were administered as follows: 43 (84.3%) patients received idarubicin and cytarabine (IA) treatment, 7 (13.7%) patients received venetoclax plus azacytidine (VA) treatment, and 1 (2.0%) patient received decitabine combined with homoharringtonine, cytarabine and granulocyte colony stimulating factor (G-CSF) (DHAG) treatment. After induction therapies, measurable residual disease (MRD) negative and positive were in 38 (74.5%), and 13 (25.5%) patients, respectively. Additional characteristics of the enrolled patients are summarized in Table 1.

### Response to therapy

**Fig. 2:**
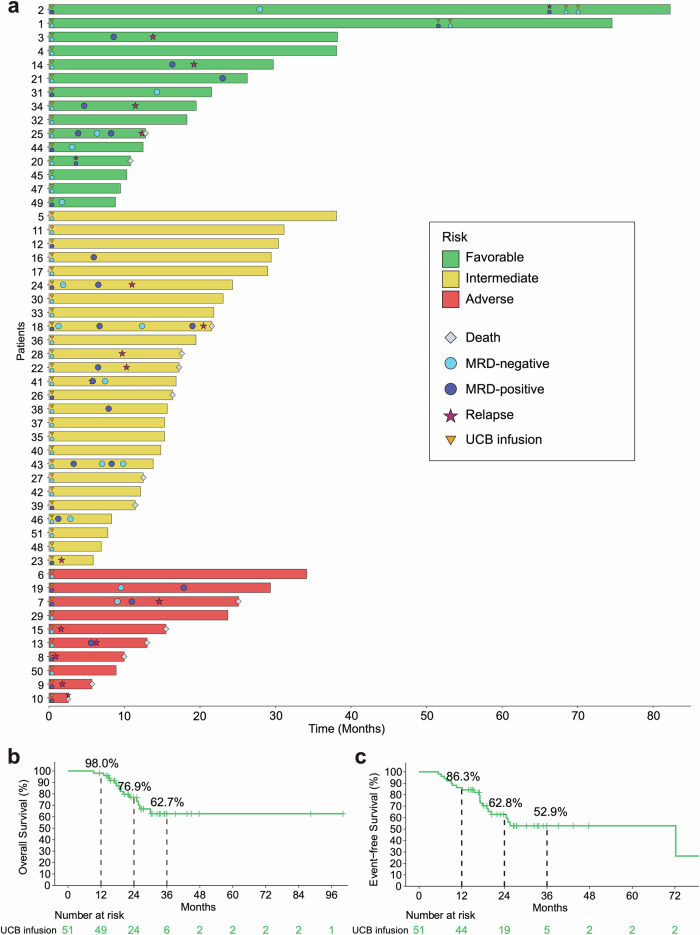
Survival outcomes in patients treated with UCB infusion as an adjuvant consolidation therapy. **a** Swimmer plot of dynamic response for patients following two cycles of UCB infusion treatment (*n* = 51). Each bar represents an individual patient with ELN risk group color-coded. Different symbols indicate different events, with MRD labeled at the time of UCB infusion and upon the status changed. **b** Kaplan-Meier curves of OS for patients treated with UCB infusion (*n* = 51). The median OS for UCB infusion was not reached (range, 9.3–100 months). **c** Kaplan-Meier curves of EFS for patients treated with UCB infusion (*n* = 51). The median EFS for UCB infusion was 72.2 months (range, 5.4–100 months)

All patients achieved the first CR (CR1) following induction therapies and completed the planned 2 cycles of chemotherapy combined with UCB infusion as an adjuvant consolidation therapy. Upon the final follow-up (31 March 2023), all patients had been followed-up for at least 1 year or met the primary endpoint. Thirty-three (64.7%) patients remained in CR, while 18 (35.3%) patients experienced relapse (Fig. 2a, Table 2). The continued CR occurred in 9 (9/15, 60.0%), 20 (20/26, 76.9%) and 4 (4/10, 40.0%) patients with ELN favorable, intermediate and adverse risks, respectively (Fig. 2a, Table 2, Supplementary Fig. 1a). Thirty-seven patients (72.5%) were alive, and 14 patients (27.5%) died (Fig. 2a, Table 2, Supplementary Fig. 1b). Among the 14 deaths, 11 patients died of relapsed AML. Three patients died of AML unrelated diseases, of whom 2 patients died of SARS-CoV-2, and 1 patient died of Burkitt lymphoma (Supplementary Table 1). No significant difference was observed in the distribution of MRD status before and after consolidation (*P* > 0.05, Supplementary Fig. 2).

**Table 2:** Response summary

Endpoint	Overall (*n* = 51)	Favorable risk (*n* = 15)	Intermediate risk (*n* = 26)	Adverse risk (*n* = 10)
Relapse, *n* (%)	18 (35.3)	6 (40.0)	6 (23.1)	6 (60.0)
Death, *n* (%)	14 (27.5)	2 (13.3)	6 (23.1)	6 (60.0)
Neutrophils ≥0.5 × 10^9^/L, median (range), days	12.2 (0–21)	11.9 (0–18)	11.9 (6–18)	13.5 (10–21)
Platelets ≥20 × 10^9^/L, median (range), days	11.5 (6–17)	11.2 (6–15)	11.7 (7–17)	11.7 (8–16)
Platelets ≥50 × 10^9^/L, median (range), days	14.8 (6–22)	14.3 (6–21)	15.1 (7–22)	15.1 (10–21)
OS, median (range), months	NR (9.3–100)	NR (14.8–100)	NR (11.3–43.2)	20.1 (9.3–39.1)
EFS, median (range), months	72.2 (5.4–100)	72.2 (7.7–100)	NR (7.1–43.2)	15.8 (5.4–39.1)

*OS* overall survival, *EFS* event-free survival, *NR* not reached

The median time to neutrophil recovery (neutrophils ≥0.5 × 10^9^/L) was 12.2 days (range, 0 to 21 days) (Table 2). The median times to recovery of platelets ≥ 20 × 10^9^/L and 50 × 10^9^/L were 11.5 days (range, 6 to 17 days) and 14.8 days (range, 6 to 22 days) (Table 2). The median OS was not yet reached (NR) and the median EFS was 72.2 months (range, 5.4–100 months) at a median follow-up of 27.3 months (range, 9.3–100 months) (Table 2). This regimen demonstrated favorable outcomes, with the OS rates at 1-, 2- and 3-year of 98.0%, 76.9%, and 62.7%, respectively. The EFS rates at 1-, 2- and 3-year were 86.3%, 62.8%, and 52.9%, respectively (Fig. 2b, c, Supplementary Table 1). There was a trend that MRD negative patients displayed better survival, though statistical significance was not reached (Supplementary Fig. 3). Survival outcomes stratified by ELN risk groups are illustrated in Supplementary Fig. 4.

### Safety profile

All patients enrolled in this trial were included in the toxicity analysis. The treatment-related adverse events (AEs) by different types and grades during post-remission consolidation therapy are summarized in Table 3. All (100%) patients encountered at least one treatment-related AE attributed to the chemotherapy treatment; however, no grade 5 AEs were observed.

**Table 3:** All treatment-related AEs by type and grade

Event, *n* (%)	All Grades	Grade 1–2	Grade 3–4
Hematological Toxicity			
Thrombocytopenia	31 (60.8)	2 (3.9)	29 (56.9)
Neutropenia	31 (60.8)	12 (23.5)	19 (37.3)
Anemia	30 (58.8)	24 (47.1)	6 (11.8)
Neutropenic fever	7 (13.7)	5 (9.8)	2 (3.9)
Non Hematological Toxicity			
Mucositis	6 (11.8)	6 (11.8)	0
Skin disorders	4 (7.8)	4 (7.8)	0
Cardiac disorders	3 (5.9)	3 (5.9)	0
Hepatobiliary disorders	1 (2.0)	1 (2.0)	0
Sepsis	1 (2.0)	1 (2.0)	0
aGVHD/cGVHD	0	0	0

*aGVHD* acute graft-versus-host disease, *cGVHD* chronic acute graft-versus-host disease

The most frequent hematological toxicities (all grades) were thrombocytopenia (60.8%), neutropenia (60.8%) and anemia (58.8%). Specifically, 2 (3.9%) patients demonstrated grade 1–2 thrombocytopenia, and 29 (56.9%) suffered grade 3–4 thrombocytopenia. Twelve (23.5%) patients had grade 1–2 neutropenia while 19 (37.3%) suffered grade 3–4 neutropenia. Twenty-four (47.1%) patients encountered grade 1–2 anemia, and 6 (11.8%) experienced grade 3–4 anemia.

The most common non-hematologic AEs were mucositis (6/51, 11.8%), skin disorders (4/51, 7.8%) and cardiac disorders (3/51, 5.9%) in grade 1–2. No grade 3–4 non-hematologic toxicities occurred in any patient. No cases of acute or chronic GVHD (aGVHD/cGVHD) or toxicity-related death were reported in any patient.

### Single-cell transcriptome exploratory analysis

**Fig. 3:**
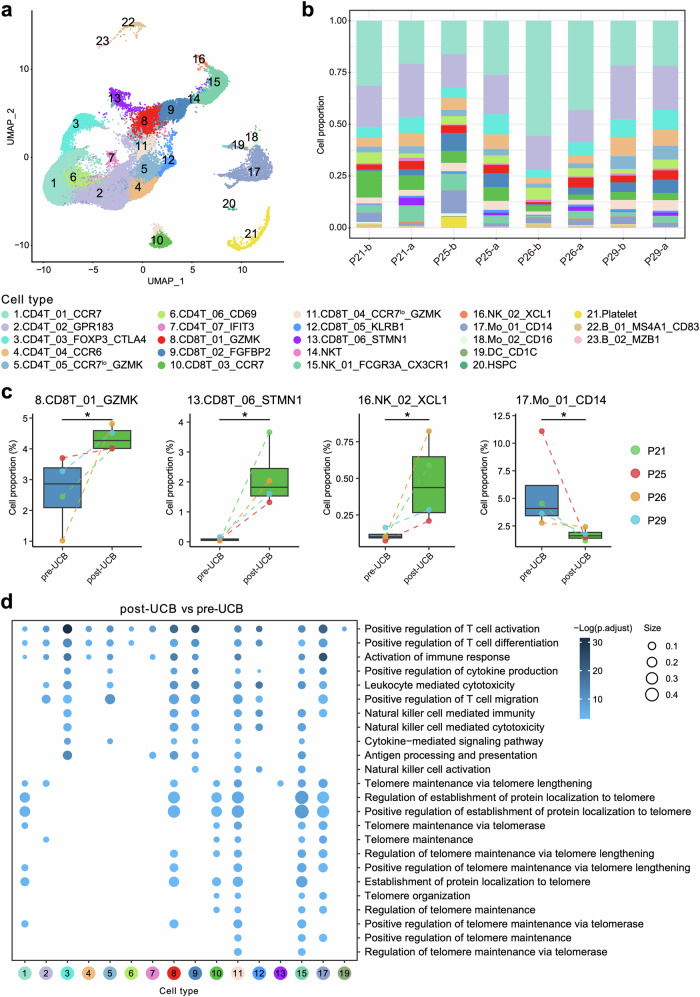
Patients’ responses by single-cell analysis of matched peripheral blood samples from patients collected at 1 day before and 7 days after UCB infusion. **a** Uniform manifold approximation and projection (UMAP) plot depicting the 23 identified cell types. Each dot represents an individual cell. Cell populations are coded by different colors. CD4T CD4^+^ T cells, CD8T CD8^+^ T cells, NKT nature killer/T cells, NK natural killer cells, Mo monocyte cells, DC dendritic cells, HSPC hematopoietic stem and progenitor cells. **b** Stacked bar blot showing the proportions of the 23 identified cell types across each sample. The colors indicate different cell populations as same as panel a. P represents patients, b represents before UCB infusion and a represents after UCB infusion. **c** Box plot illustrating the dynamic changes in cell fractions of cytotoxic T cells (CD8T_01_GZMK, CD8T_06_STMN1), NK cells (NK_02_XCL1) and monocytes (Mo_01_CD14). The colors represent different patients and time points. * denotes statistical significance (*P* < 0.05) performed by using the Wilcoxon rank sum test. **d** Dot plot visualizing the enriched GO terms of up-regulated DEGs in different cell populations post-UCB compared to pre-UCB infusion. The size of nodes represents the ratio of DEGs that are annotated in each term to all genes in that term. Colors represent the adjusted one-sided Fisher’s exact test *P* values by Benjamini-Hochberg correction

To elucidate the cellular and molecular mechanisms underlying UCB infusion adjuvant consolidation therapy against AML, we conducted scRNA-seq of matched pre- and post-treatment peripheral blood samples with UCB infusion using 10x technology from 4 patients (P21, P25, P26 & P29). Of these, P21 and P25 were classified into favorable risk group, of whom P25 died of relapsed AML. P26 and P29 belonged to intermediate and adverse risk groups, respectively. P26 died of AML unrelated disease from SARS-CoV-2 (Supplementary Table 1). Samples were collected at 1 day before UCB infusion (b, pre-UCB) and 7 days after UCB infusion (a, post-UCB), capturing transitions in cellular dynamics. After stringent quality control measures (Methods), we obtained transcriptome profiles of 57,279 high-quality cells (Supplementary Table 2) and identified 23 distinct cell types, including 7 CD4^+^ T, 6 CD8^+^ T, 2 natural killer (NK), 2 monocyte, 1 dendritic cell (DC) and other clusters (Fig. 3a, Supplementary Fig. 5, Supplementary Fig. 6a, and Supplementary Table 3).

We analyzed the fractions of individual cell types in scRNA-seq data to examine their contributions to the post-remission consolidation therapy responses with UCB infusion. Notably, cytotoxic CD8^+^ T cells (CD8T_01_GZMK, CD8T_06_STMN1) and NK cells (NK_02_XCL1) were increased, while CD14^+^ monocytes (Mo_01_CD14) were decreased significantly after UCB infusion (Fig. 3b, c, Supplementary Tables 4, 5, *P* < 0.05). The CD8T_01_GZMK subset, characterized by high expression of *GZMK* and *NKG7* (Supplementary Fig. 6a), was a cluster of effector CD8^+^ T cells.^26^ The CD8T_06_STMN1 subset, expressed high levels of proliferation makers *STMN1* and *MKI67* (Supplementary Fig. 6a), representing proliferating cytotoxic CD8^+^ T cells.^27^ Developmental trajectory analysis using Monocle2^28^ indicated a clear sequential differentiation path from naïve CD8^+^ T (CD8T_03_CCR7) transitioning into early effector CD8^+^ T (CD8T_04_CCR7^lo^_GZMK), followed by effector memory CD8^+^ T (CD8T_02_FGFBP2, CD8T_05_KLRB1), and ended with cytotoxic and proliferating effector CD8^+^ T cells (CD8T_01_GZMK, CD8T_06_STMN1) (Supplementary Fig. 6b, c). This is in line with that CD8^+^ effector T cells continue to engage in antigen specific interactions, driving further proliferation and cytokine release.^29^ The NK_02_XCL1 cells expressed high levels of *NKG7*, *GNLY* and *XCL1* (Supplementary Fig. 6a), which are crucial for recruiting DCs to enhance anti-tumor immunity.^30^ Conversely, the decreased fraction of Mo_01_CD14, a classical inflammatory monocyte cluster (Supplementary Fig. 6a) associated with tumor progression and immunosuppression,^31,32^ suggested a shift towards less tumor-promoting activity after UCB infusion.

Gene Ontology (GO)^33^ analysis of differentially expressed genes (DEGs) highlighted significant enrichment in processes such as antigen processing and presentation, activation of immune response, positive regulation of T cell activation, migration and differentiation, positive regulation of cytokine production, leukocyte mediated cytotoxicity, etc., after UCB infusion (Fig. 3d). Interestingly, telomere maintenance via telomerase or telomere lengthening, and positive regulation of establishment of protein localization to telomere functions were also enhanced (Fig. 3d), suggesting a potential anti-aging effect of UCB infusion.

These findings collectively indicate that UCB infusion promotes cell proliferation and cytokine secretion of CD8^+^ T cells and NK cells that enhance their immune functions, and mitigates the immunosuppressive effects of CD14^+^ monocytes. Meanwhile, addition of UCB enhances telomerase activity and telomere stabilization, which in turn exerts anti-aging effects. Overall, our single-cell analyses underlined the anti-tumor and anti-aging benefits in elderly AML patients treated with UCB infusion as an adjuvant consolidation therapy.

## Discussion

This phase 2 trial revealed encouraging clinical responses to UCB infusion in elderly patients with AML, resulting in 64.7% (33/51) durable CR after post-remission therapy, with a median follow-up of 27.3 months (range, 9.3–100 months). At the last follow-up, 37 patients (72.5%) were alive, while 14 patients (27.5%) had died. Among these deaths, 11 patients died of relapsed AML. Three patients who remained in CR died of AML unrelated diseases, of whom 2 patients died from SARS-CoV-2, and 1 patient died from Burkitt lymphoma afterwards. Notably, the median OS was yet not reached and the median EFS was 72.2 months (range, 5.4–100 months). The OS rates at 1-, 2- and 3-year were 98.0%, 76.9%, and 62.7%, respectively, with corresponding EFS rates of 86.3%, 62.8%, and 52.9% for UCB infusion treatment.

In contrast, CR in elderly AML patients following intensive chemotherapy typically results in a median EFS of 6 to 12 months, and only about a 10% 5-year OS rate from diagnosis.^34,35^ Numerous efforts have been tried to investigate the value of post-remission chemotherapy in aged AML patients. For instance, cytarabine, used in various dose schemes, did not produce benefit in the management of AML for elderly patients after CR.^36–38^ Gemtuzumab ozogamicin as post-remission therapy in AML patients with 60 years old or more also did not produce prognosis benefits, with the OS and EFS rates at 2-year of 45 and 34%, respectively.^35^ Another study reported the 1-, 2-, and 3-year EFS rates were 64%, 44 and 32%, respectively, by using azacytidine as post-remission treatment in AML patients over 60 years.^34^ Our regimen had a clear survival benefit with the OS and EFS rates at 2 years of 76.9% and 62.8%, respectively, in the context of previous studies on post-remission therapies in elderly AML patients.

Additionally, a previous study investigated the efficacy of decitabine plus intermediate dose cytarabine combined with HLA-mismatched G-CSF-mobilized peripheral blood stem cells infusion (D-GPBSCs regimen) in 23 AML patients aged ≥ 60 years old in CR1. In that cohort, the OS and EFS rates at 2-year were 55.4% and 51.6% in D-GPBSCs group, respectively, with the median times to platelet ( ≥ 20 × 10^9^/L) and neutrophil recovery of 14 and 12 days, respectively.^39^ In comparison, we reported here on a larger and a longer follow-up cohort treated with decitabine plus intermediate dose cytarabine followed by UCB infusion as an adjuvant consolidation therapy, revealing improved 2-year OS (76.9% vs. 55.4%) and EFS (62.8% vs. 51.6%) rates over previous D-GPBSCs regimen.

For allo-HSCT post-remission treatment, Kadia and colleagues presented a phase 2 study of the addition of venetoclax to a regimen of cladribine and low dose cytarabine, alternating with 5-azacitidine (CLAD/LDAC/Ven) in elderly AML patients.^40^ They reported a 2-year OS rate of 63.5% with 34% (19/56) of patients undergoing allo-HSCT.^40^ Our regimen had a favorably improved 2-year OS rate of 76.9% without allo-HSCT. However, it should be noted that CLAD/LDAC/Ven regimen broadens the population of patients who may prefer allo-HSCT as post-remission therapy.^41^

scRNA-seq analysis demonstrated favorable immunological changes between pre- and post-UCB infusion samples. Observation in increased fractions of cytotoxic and proliferative CD8^+^ T cells in elderly patients with AML suggests their pivotal roles in anti-tumor immune response by addition of UCB as an adjuvant therapy. NK cells also have a vital role in anti-tumor activities. NK cells derived from UCB typically display an immature phenotype, however, they can acquire potent cytotoxicity with phenotypic maturation.^21^ In present study, we discovered an increase of NK cells subset characterized by high expression of *NKG7*, *GNLY* and *XCL1* following UCB infusion. *XCL1* has been previously reported to mediate recruitment of DC to promote anti-tumor immunity.^42^ These findings indicate the NK_02_XCL1 cell population may contribute to the killing of leukemia cells after UCB infusion. Further in-vitro studies are warranted to explore their target ligands by isolating these immunologic cells from patients and co-culturing them with leukemic cells.

Moreover, UCB infusion was not only associated with anti-tumor effects, but also with active anti-aging benefits, characterized by telomere synthesis/maintenance via activation of telomerase and telomere associated proteins. Telomere length maintenance is significant for preventing T cells from undergoing senescence.^43^ Upregulation of telomerase activity is positively related to activation induced T cells proliferation and viability, thereby protecting them from apoptosis.^44^

Cancer is often considered as a disease of aging and most of cancers occur over 60 years old.^45^ Aging, the major risk factor for cancer, influences every aspect of cancer pathogenesis, from premalignant growths to disease progression, and to therapeutic responses. It underlies declined functions of recognizing and eliminating tumor cells as well as more immunosuppressive activities.^45^ Anti-aging therapy or combined therapies with anti-aging treatment might be a new therapeutic dimension that rejuvenates immune system to systematically fight against cancer.

Noted limitations of our study are the limited number of patients, relative short follow-up and no other participated centers. A prospective, randomized, phase 3 confirmatory trial with more patients and longer follow-up and multi-center will be carried out to further confirm our observations in this study. Two time points and a small number of patients in the exploratory analyses should be also noted. More time points and larger sample size of correlative analyses may help interrogate markers of remission, early relapse or MRD recurrence. Despite these limitations, this phase 2 pilot trial with favorable outcomes helps inform the design of randomized controlled trial, and lays the groundwork for further assessment of the impact of UCB infusion.

In conclusion, our findings demonstrate the favorable clinical outcomes of UCB infusion as an adjuvant consolidation regimen for elderly patients with AML, and shed light on the mechanisms of immunological changes that are associated with the improved prognosis. Based on these encouraging findings, we believe that UCB infusion as a post-remission adjuvant therapy is highly active, well tolerated and warrants further investigations in elderly patients with AML.

## Materials and methods

### Study design and participants

This was a prospective, single-arm, open label, phase 2 study evaluating UCB infusion as an adjuvant consolidation regimen in elderly AML patients (ChiCTR-OPC-15006492). This trial was approved by the Human Ethics Committee of Ruijin Hospital affiliated to Shanghai Jiao Tong University School of Medicine (RJ-201646). The protocol was in accordance with Declaration of Helsinki Principles and International Conference on Harmonization (ICH) Good Clinical Practice (GCP) Guidelines. After the institutional review board approval, the trial recruited patients at Ruijin Hospital. Written informed consents were obtained from all enrolled patients.

The complete inclusion criteria are shown below: age ≥60 years old; newly diagnosed with de novo AML, examined by bone marrow according to the WHO 2022 criteria; ECOG performance score 0 to 2; echocardiography examination of Left Ventricular Ejection Fraction (LVEF) ≥ 50%; estimated glomerular filtration rate (eGFR) ≥ 60 mL/min, measured using the CKD-EPI formula; alanine aminotransferase (ALT) ≤ 2.5×upper limit of normal (ULN), aspartate aminotransferase (AST) ≤ 2.5×ULN and total bilirubin ≤ 1.5×ULN; documented CR after one or two cycles of induction therapies defined according to standard criteria; inability or unwillingness to undergo allo-HSCT; signed informed consent before admission to the study.

The complete exclusion criteria are shown below: acute promyelocytic leukemia (APL); secondary AML (sAML); subjects who had been pretreated with other investigational drugs and/or currently participate in any other clinical trials; subjects with known involvement of central nervous system (CNS); subjects with known history of human immunodeficiency virus (HIV) infection; subjects with known history of hepatitis B virus (HBV) or hepatitis C virus (HCV) infection; New York Heart Association (NYHA) functional classification higher than grade 2; subjects with chronic respiratory disease requiring continuous oxygen inhalation; subjects with other malignant tumors or hematological system diseases; subjects with uncontrolled systemic infection (viral, bacterial or fungal); subjects with known or suspected autoimmune diseases; subjects with known history of intolerance or allergy to congeneric drugs; inability or unwillingness to follow the required protocol procedures; familial, psychological, geographical or sociological factors potentially impeding compliance with the protocol procedures and follow-up schedules; any uncontrolled or serious medical disorders that, at the discretion of the investigators, may increase the risks related to study participants or drug administrations, impair the abilities of the patients to undergo protocol therapies or obstruct the data interpretation of the study.

The first patient was enrolled on 12 January 2015 and the last patient on 12 February 2022. Written informed consents were received from all participants or their legal representatives, before any study related tests or procedures. There were no protocol amendments during the conduct of the study. Data were collected by the investigators and analyzed by the authors. The authors are committed to the fidelity of the trial, and the accuracy and integrity of the data in the study protocol.

### Treatment protocol

The post-remission treatments for elderly patients with AML consisted of two cycles of 15 mg/m^2^ low dose intravenous decitabine over 4 h per day for consecutive 5 days (day 1–5), 1.0 g/m^2^ intermediate-dose cytarabine at q12h for consecutive 2 days (day 6–7), combined with one unit UCB infusion on day 9 as an adjuvant consolidation therapy. High resolution HLA typing of HLA-A, HLA-B and HLA-DR loci was carried out for all enrolled participants. The UCB units were gained from Shandong Province Cord Blood Bank (SINOCORD), if they were serologically matched of at least 4 HLAs and contained more than 3 × 10^7^ nucleated cells per kilogram of patient’s body weight before freezing.

No immunosuppression was given as prophylaxis for GVHD, unless aGVHD was documented or diagnosed clinically. Infection prophylaxis or other support therapies, for example G-CSF, were administered following the regular transplantation procedure. The hematopoietic cell transplantation comorbidity index (HCT-CI) was applied to evaluate the comorbidities prior to each cycle of consolidation therapy. The second cycle of consolidation therapy was repeated after bone marrow recovery. After two cycles of consolidation therapy, patients received demethylation treatment as maintenance therapy. No targeted drugs were used in this study due to the lack of availability.

Patients underwent medical history inquiry, physical examinations, and molecular profiling at enrollment. Laboratory tests, including coagulation parameters, complete blood count, urine protein and urinalysis, a full set of biochemistries, electrocardiogram (ECG), echocardiography, abdominal ultrasound scan, chest CT scan, karyotyping, cytological and MRD detection of bone marrow biopsy, were performed at baseline and periodically thereafter at the treating investigators’ discretion. Response was assessed after each cycle of consolidation. Follow-up assessments were carried out monthly for the first year, and every 3 months thereafter.

Leukemia associated immunophenotyping (LAIP) was determined at diagnosis by using different cell surface markers. Bone marrow cytology and MRD were assessed according to the previously described process by muti-parameter flow cytometry.^46^ The monoclonal antibodies against 20 antigens were as follow: CD2, cyCD3, CD4, CD7, CD11b, CD13, CD14, CD15, CD19, CD33, CD34, CD38, CD45, CD56, CD64, cyCD79a, CD117, HLA-DR, MPO, and TdT. MRD negativity was defined as MRD < 0.01%. The samples were considered as evaluable if they contained 100,000 cells or more. Otherwise, they were determined as unevaluable.

On the day 7 after UCB infusion, peripheral blood cells were collected from all patients and assessed for chimerism using standard cytogenetic and a semi-quantitative polymerase chain reaction based assay of short tandem repeats with the sensitivity of 1%.

### Study endpoints

The primary endpoint of this trial was OS, defined as the time interval from the date of diagnosis to the date of death from any cause, with censoring of patients known to be alive upon the last follow-up. Secondary endpoints included EFS, bone marrow MRD by flow cytometry, treatment-related AEs, and the times to platelet and neutrophil count recovery. EFS was determined as the time interval from the date of diagnosis to the date of occurrence of any following events, including relapse or death, whichever came first. MRD relapse was not considered as an event for EFS, as not all MRD positive patients end with relapse.^9^ NRM was defined as death during continuous CR1. Exploratory endpoint was blood-based biological characteristics exploratory study analyzed by scRNA-seq of matched pre- and post-UCB infusion samples.

### Evaluation of safety

All the patients enrolled were included in the safety analyses. Treatment-related AEs, including hematological and non-hematological AEs, were defined as those that occurred from the start of treatment. Early deaths were defined as deaths occurring within 30 days of treatment with this regimen. The severity of AEs was assessed following the Common Terminology Criteria for Adverse Events (CTCAE, v5.0) of National Cancer Institute during treatment. AEs monitoring began after the first patient enrollment and was monitored continuously until the last follow-up. Grade 1–2 were defined as mild events, while grade 3–4 were defined as severe events.

### Single-cell library preparation and sequencing

The cell suspension (cell viability >80%, measured by Count Star) was processed using the Chromium single-cell controller (10x Genomics) to produce single-cell gel beads in emulsion, based on the manufacturer’s instructions. The quality of complementary DNA (cDNA) was evaluated using an Agilent 4200 system (Agilent Technologies). The libraries were then prepared utilizing the Chromium Single Cell 5’ Reagent Kits (10x Genomics, v1.1), according to the manufacturer’s protocol. The single-cell libraries were run on a MGISEQ 2000 sequencer (MGI) at the sequencing platform of National Research Center for Translational Medicine at Shanghai.

### Single-cell sequencing data analysis

Sequencing data were demultiplexed and converted to FASTQ format files using spliteBarcode (MGI, v2.0.0) software. The raw reads were then mapped to the GRCh38 reference genome using cellranger (10x Genomics, v5.0.1) toolkit. In particular, cellranger count was employed to process the gene expression library sequencing data and generate the gene-barcode matrix.

The Seurat (v4.0.2) package in R (v4.1.0) was used for subsequent analyses.^47–49^ For quality control, the cells with percentage of mitochondrial genes more than 50%, detected genes less than 200 or more than 6000, were excluded from the analysis. Filtered data were log normalized using a scaling factor of 10,000. The most 2000 variable features per sample were selected using the variance stabilizing transformation (vst) method. To account for the batch effects across different samples, we utilized the canonical correlation analysis (CCA) approach within the Seurat package for data integration. The batch corrected and scaled data were used for dimensionality reduction.

Principal component analysis (PCA) was carried out to reduce the CCA merged data to the top 50 principal components (PCs). The first 30 PCs were utilized for further analysis based on the ElbowPlot. Clustering were identified using the shared nearest neighbor (SNN) graph-based model in the function FindClusters with resolution = 0.8. The same PCs (top 30) were utilized to generate Uniform Manifold Approximation and Projection (UMAP)^50^ for data visualization. Clusters were annotated by the expression patterns of canonical marker genes.

To identify the differentially expressed markers for each cluster, we utilized Wilcoxon rank sum test embedded in the functions FindAllMarkers and FindMarkers for multiple and two condition comparisons, respectively. Significant differentially expressed markers were identified as those with log2 fold change of average expression larger than 0.25, adjusted *P* values by Bonferroni-Hochberg less than 0.05, and expression present in more than 10% of cells. Gene ontology (GO) enrichment analysis was carried out by utilizing clusterProfiler (v4.6.2).^51^

Single cells assigned to CD8^+^ T cell clusters from all samples were employed for pseudotime analysis and diffusion map. We applied Monocle2 (v2.22.0) for the trajectory analysis with using the parameters of percentage of cells expressing each feature larger than 0.1 and genes expressed in at least 100 cells.^28^ The DEGs for each cluster were determined based on a likelihood ratio test. The significantly DEGs identified as those with adjusted *P* values less than 0.01 were selected as the ordering genes for trajectory reconstruction using the DDRTree nonlinear reconstruction algorithm.

### Statistical analysis

Clinicopathological characteristics of patients were summarized by utilizing frequencies (%) for categorical variables and medians (range) for continuous variables. Fisher’s exact test was used to compare the associations for categorical variables versus categorical variables. Wilcoxon rank sum test was employed to compare categorical variables versus continuous variables. False discovery rate (FDR) corrected by Benjamini-Hochberg approach was applied to adjust the *p* value for multiple testing unless otherwise specified. Kaplan–Meier survival curves were utilized to evaluate the probabilities of OS and EFS. Log-rank test was performed to compare the survival curves. Asterisks define significance levels. All statistical tests were two-sided unless otherwise specified. Statistical analyses were carried out using R (v4.1.0) packages.

## Supplementary information

Supplementary Materials

Clinical Study Protocol

## Source data

Source data

## Data Availability

No custom codes were developed for this project. All statistical packages and codes used in this study are available upon request.

## Data availability

The raw and processed scRNA-seq data produced in this work have been archived in the National Omics Data Encyclopedia (NODE), with the accession code OEP005257. Data are under controlled access due to the conditions stipulated in the patient consent process. Data can be accessed from the corresponding author upon written request and detailed proposal as to how the data will be used from the requestor. All requests will be reviewed based on departmental and institutional policies, according to the Regulation on the Administration of Human Genetic Resources of the People’ Republic of China. Requests will be responded to within 2 weeks. The GRCh38 reference genome can be assessed at https://www.10xgenomics.com/cn/support. Source data are provided in this paper. Other data are available as Supplementary Information within this article. All data shared are de-identified.
