# Supplementary material for: A phase 2 pilot study of umbilical cord blood infusion as an adjuvant consolidation therapy in elderly patients with acute myeloid leukemia
Source: Signal Transduct Target Ther. 2024 Dec 20;9:358. doi: 10.1038/s41392-024-02065-y (PMC11659310; doi:10.1038/s41392-024-02065-y)
Supplement: Supplementary file 1 — Supplementary Materials [file 41392_2024_2065_MOESM1_ESM.docx]

Supplementary Materials for

**A phase 2 pilot study of umbilical cord blood infusion as an adjuvant consolidation therapy in elderly patients with acute myeloid leukemia**

Jinzeng Wang^1,#^, Xiaoyang Li^1,#^, Ping Liu^1,#^, Yao Dai^1^, Hongming Zhu^1^, Yunxiang Zhang^1^, Min Wu^1^, Yunying Yao^1^, Mingzhu Liu^1^, Shuting Yu^1^, Fangying Jiang^1^, Shuai Wang^1^, Haoran Mu^2^, Bo Jiao^1^, Hua Yan^1^^,3^, Wen Wu^1^, Yang Shen^1^, Junming Li^1,*^, Shengyue Wang^1,*^, Ruibao Ren^1,4,5,*^.

# These authors contributed equally to this work

Correspondence to : [rbren@sjtu.edu.cn](mailto:rbren@sjtu.edu.cn); [wsy12115@rjh.com.cn](mailto:wsy12115@rjh.com.cn); [drlijunmin@126.com](mailto:drlijunmin@126.com).

**This PDF file includes:**

Figures. S1 to S6

Tables S1 to S5

**
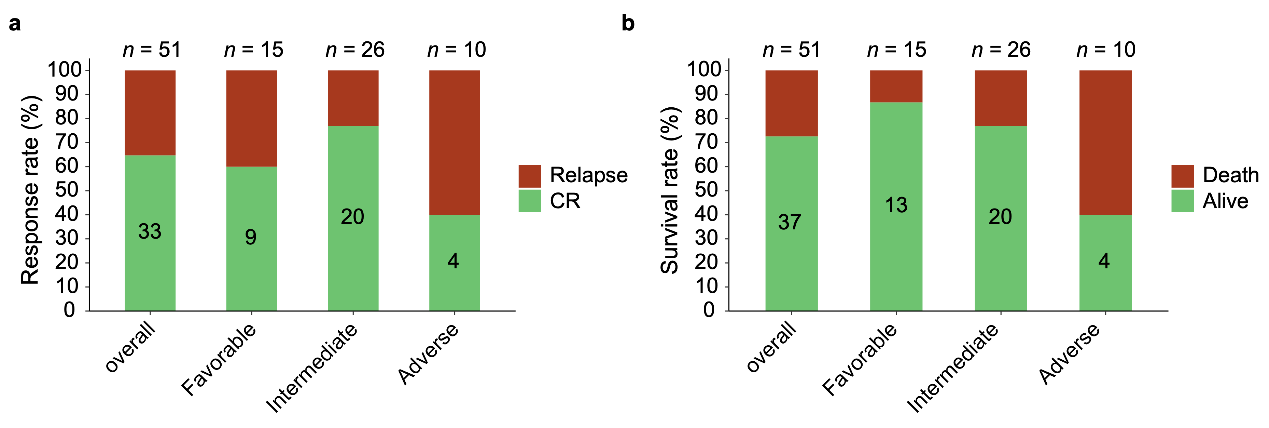
**

**Figure. S1. Patient responses to UCB infusion as an adjuvant consolidation therapy. a,** Patient responses after consolidation therapy in different ELN risk groups. **b**, Patient survival after consolidation therapy in different ELN risk groups. The total number of patients in each category is illustrated above the bar, with the number of patients alive or remaining in CR labeled below.


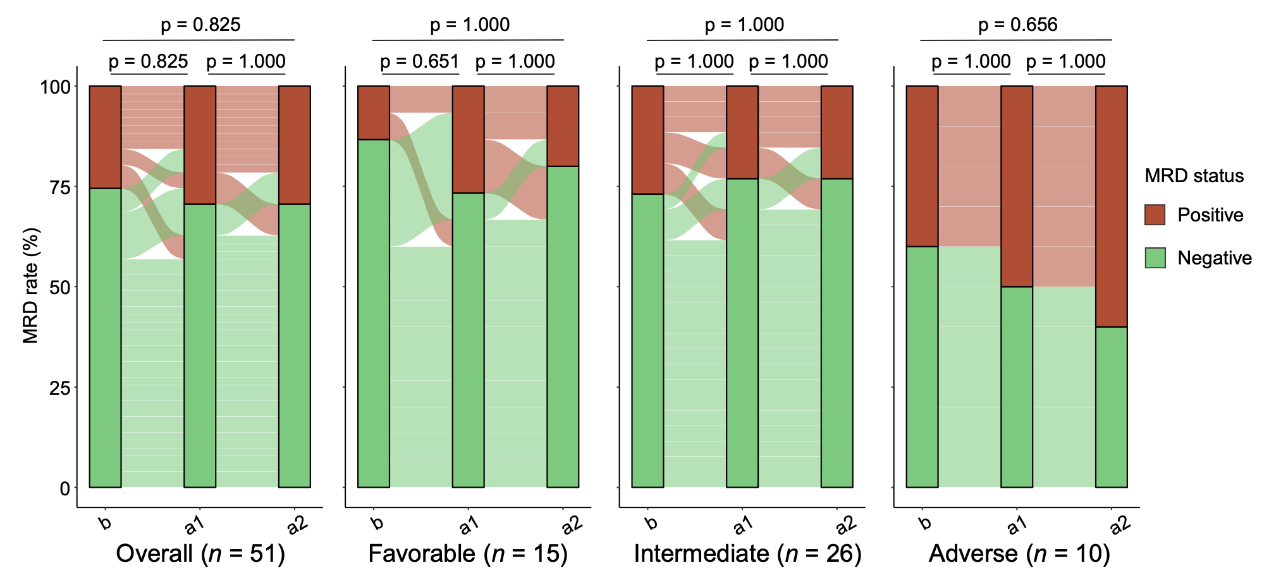


**Figure. S2. MRD status before consolidation (b), after one (a1) and two (a2) cycles of UCB infusion**. Statistical analysis was performed using the Fisher’s exact test.

**
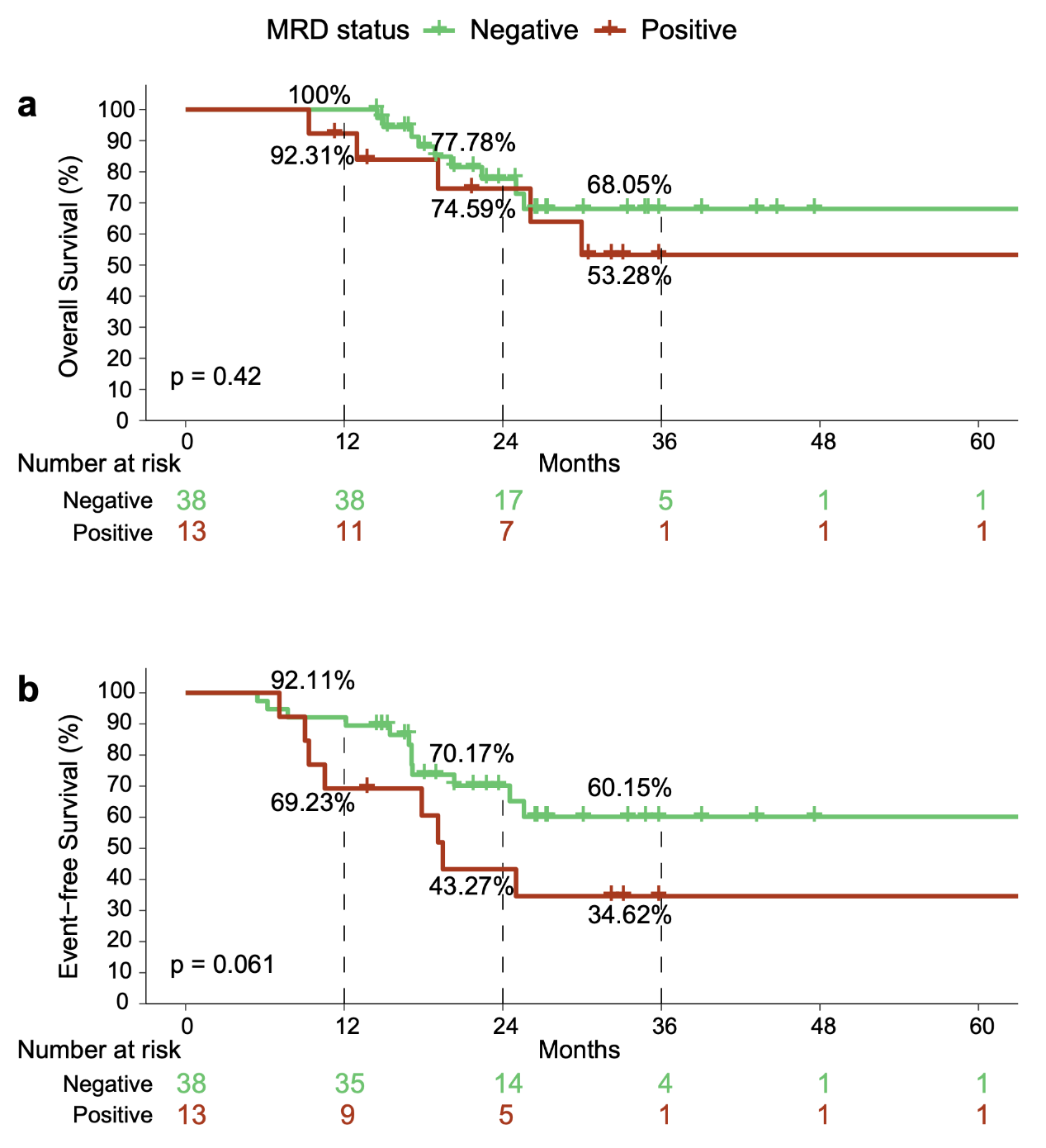
**

**Figure. S3. Impact of MRD status prior to UCB infusion as an adjuvant consolidation therapy on survival outcomes. a-b**, Kaplan-Meier curves of OS (a) and EFS (b) by MRD status. Statistical analysis was performed using the log-rank test.

**
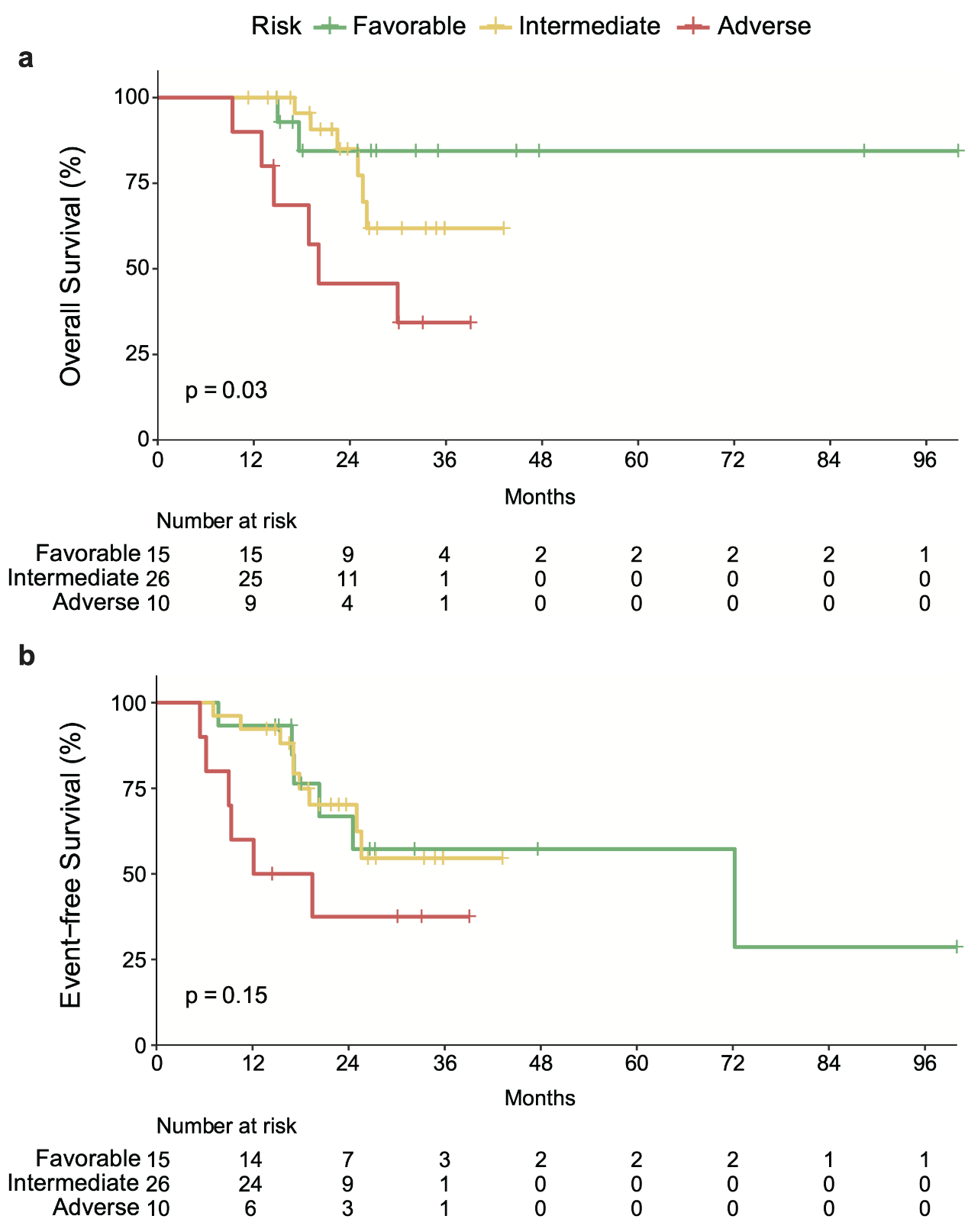
**

**Figure. S4. Impact of ELN risk on survival outcomes in patients treated with UCB infusion as an adjuvant consolidation therapy. a-b**, Kaplan-Meier curves of OS (a) and EFS (b) by ELN risk groups. Statistical analysis was performed using the log-rank test.

**
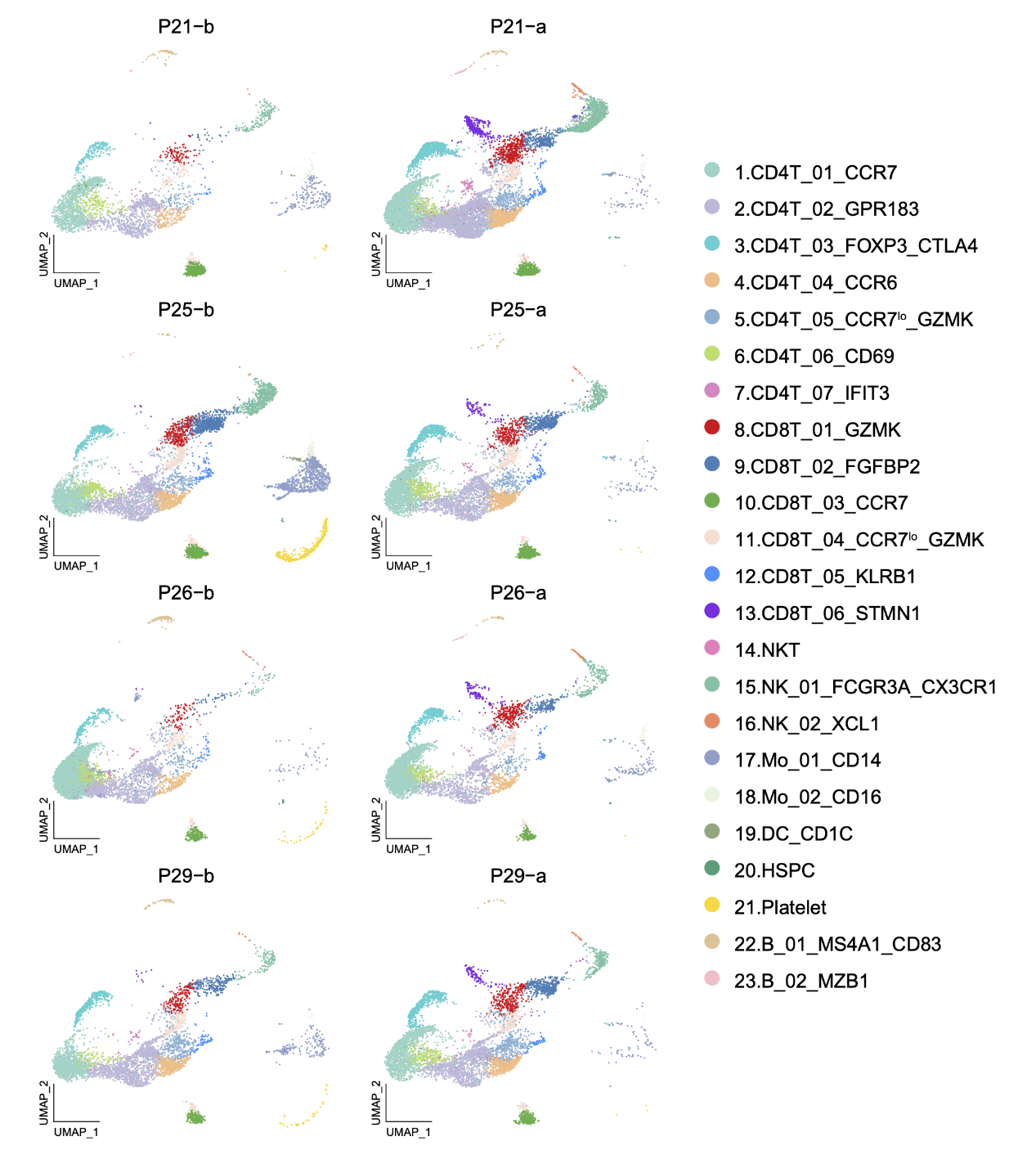
**

**Figure. S5. Clustering of peripheral blood scRNA-seq data by samples.** UMAP plots of individual samples. Each dot represent a single cell. Colors represent different cell populations.

**
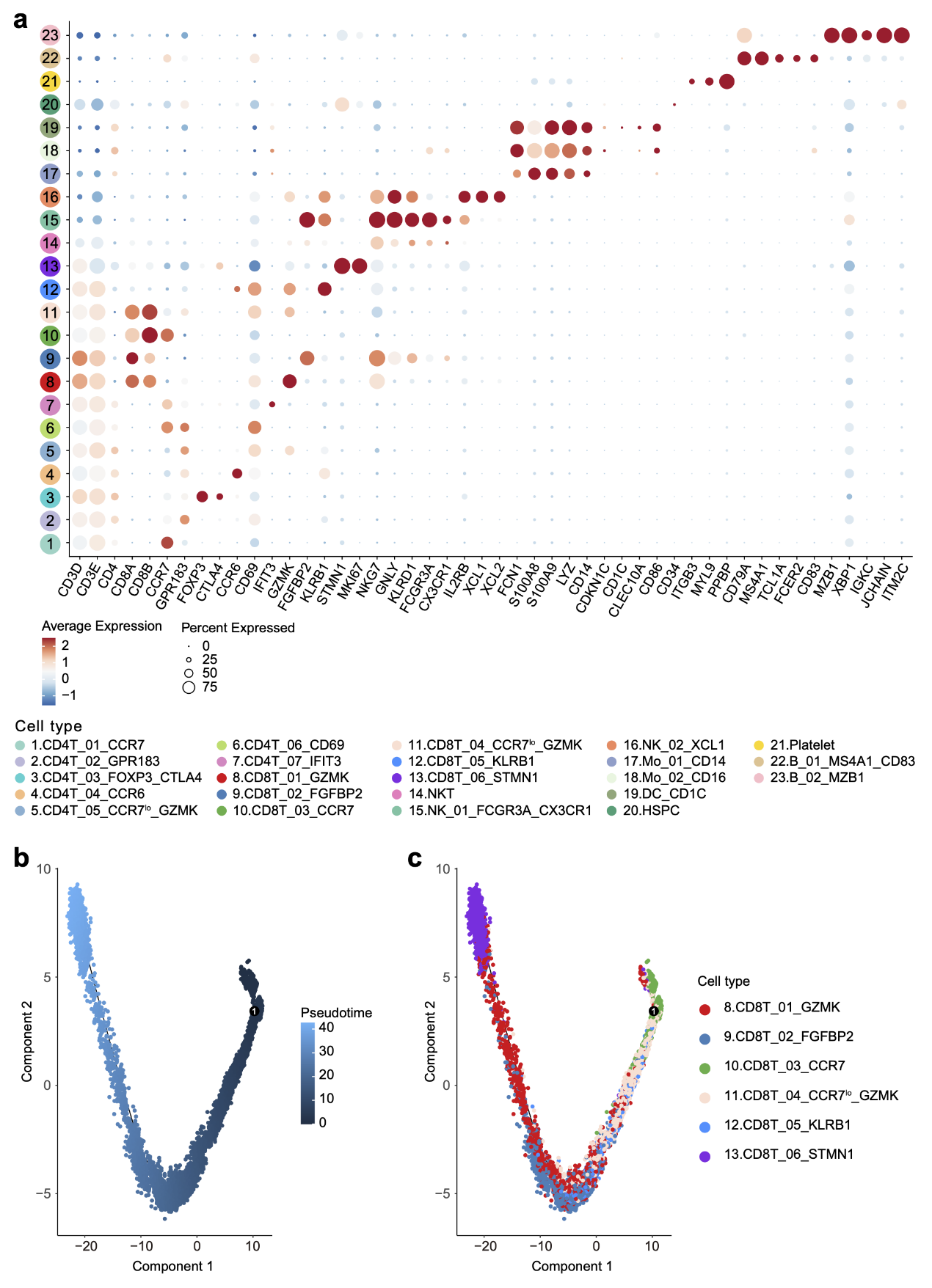
**

**Figure. S6. Features of the 23 identified cell types and the pseudotime trajectory of CD8^+^ T cells. a**, Dot plot showing the expression and abundance of canonical marker genes among the 23 cell populations. **b**, Trajectory path of CD8^+^ T cells inferred by Monocle2. Colors correspond to cluster annotations and dots represent individual cells. Cell orders are inferred based on the expression of the most variable genes across CD8^+^ T cell clusters.

**Table S1. Characteristics of enrolled AML patients with response data**

| **Patient** | **Age at Diagnosis** | **Gender** | **Relapse Status** | **Vital Status** | **OS (Days)** | **EFS (Days)** |
| --- | --- | --- | --- | --- | --- | --- |
| 1 | 63 | Female | 0 | 0 | 3000 | 3000 |
| 2 | 63 | Male | 1 | 0 | 2647 | 2167 |
| 3 | 72 | Male | 1 | 0 | 1343 | 610 |
| 4 | 71 | Female | 0 | 0 | 1428 | 1428 |
| 5 | 72 | Male | 0 | 0 | 1297 | 1297 |
| 6 | 65 | Male | 0 | 0 | 1172 | 1172 |
| 7 | 65 | Female | 1 | 1 | 899 | 584 |
| 8 | 68 | Male | 1 | 1 | 435 | 163 |
| 9 | 61 | Female | 1 | 1 | 389 | 271 |
| 10 | 68 | Female | 1 | 1 | 280 | 280 |
| 11 | 63 | Male | 0 | 0 | 1075 | 1075 |
| 12 | 62 | Male | 0 | 0 | 1074 | 1074 |
| 13 | 63 | Female | 1 | 1 | 566 | 364 |
| 14 | 64 | Female | 1 | 0 | 1051 | 736 |
| 15 | 68 | Female | 1 | 1 | 603 | 186 |
| 16 | 65 | Male | 0 | 0 | 1044 | 1044 |
| 17 | 63 | Male | 0 | 0 | 1004 | 1004 |
| 18 | 67 | Male | 1 | 1 | 783 | 750 |
| 19 | 67 | Female | 0 | 0 | 994 | 994 |
| 20 | 71 | Female | 1 | 1 | 449 | 232 |
| 21 | 68 | Female | 0 | 0 | 967 | 967 |
| 22 | 75 | Male | 1 | 1 | 673 | 464 |
| 23 | 66 | Female | 1 | 0 | 339 | 213 |
| 24 | 70 | Female | 1 | 0 | 915 | 536 |
| 25 | 69 | Female | 1 | 1 | 529 | 515 |
| 26 | 61 | Female | 0 | 1 (SARS-CoV-2) | 768 | 768 |
| 27 | 74 | Male | 0 | 1 (Burkitt lymphoma) | 513 | 513 |
| 28 | 65 | Male | 1 | 1 | 750 | 513 |
| 29 | 68 | Female | 0 | 0 | 903 | 903 |
| 30 | 73 | Female | 0 | 0 | 822 | 822 |
| 31 | 71 | Female | 0 | 0 | 819 | 819 |
| 32 | 68 | Female | 0 | 0 | 799 | 799 |
| 33 | 62 | Female | 0 | 0 | 793 | 793 |
| 34 | 60 | Male | 1 | 0 | 749 | 507 |
| 35 | 61 | Female | 0 | 0 | 711 | 711 |
| 36 | 70 | Female | 0 | 0 | 711 | 711 |
| 37 | 69 | Female | 0 | 0 | 683 | 683 |
| 38 | 64 | Male | 0 | 0 | 683 | 683 |
| 39 | 64 | Female | 0 | 1 (SARS-CoV-2) | 573 | 573 |
| 40 | 66 | Female | 0 | 0 | 654 | 654 |
| 41 | 61 | Female | 1 | 0 | 650 | 316 |
| 42 | 66 | Male | 0 | 0 | 610 | 610 |
| 43 | 65 | Male | 0 | 0 | 569 | 569 |
| 44 | 73 | Female | 0 | 0 | 543 | 543 |
| 45 | 72 | Female | 0 | 0 | 506 | 506 |
| 46 | 64 | Male | 0 | 0 | 497 | 497 |
| 47 | 62 | Male | 0 | 0 | 459 | 459 |
| 48 | 60 | Male | 0 | 0 | 446 | 446 |
| 49 | 69 | Female | 0 | 0 | 445 | 445 |
| 50 | 71 | Female | 0 | 0 | 434 | 434 |
| 51 | 64 | Male | 0 | 0 | 412 | 412 |

Relapse Status: 0-CR,1-Relapse; Vital Status: 0-Alive,1-Death

**Table S2. Single-cell library information of individual samples**

| **Sample** | **Cell No. after QC** | **Estimated Cell No.** | **Mean Reads**  **/Cell** | **Median Genes/Cell** | **No. of Reads** | **Valid Barcodes** |
| --- | --- | --- | --- | --- | --- | --- |
| P21-b | 4,043 | 4,803 | 92,268 | 1,287 | 443,166,405 | 90.00% |
| P21-a | 11,882 | 12,195 | 35,846 | 1,670 | 437,144,775 | 94.10% |
| P25-b | 8,156 | 8,570 | 55,589 | 1,491 | 476,398,973 | 93.00% |
| P25-a | 7,189 | 7,448 | 59,738 | 1,875 | 444,931,608 | 92.20% |
| P26-b | 7,869 | 8,485 | 54,983 | 1,275 | 466,533,297 | 91.60% |
| P26-a | 5,961 | 6,210 | 77,913 | 1,390 | 483,838,356 | 93.30% |
| P29-b | 5,496 | 5,928 | 71,155 | 1,423 | 421,809,962 | 92.30% |
| P29-a | 6,683 | 6,863 | 62,039 | 1,700 | 425,776,497 | 92.90% |

b, before UCB infusion; a, after UCB infusion; QC, quality control

**Table S3. Canonical markers used in identifying single-cell clusters**

| **Clusters** | **Marker Genes** |
| --- | --- |
| 1.CD4T_01_CCR7 | *CD3D, CD3E, CD4, CCR7* |
| 2.CD4T_02_GPR183 | *CD3D, CD3E, CD4, GPR183* |
| 3.CD4T_03_FOXP3_CTLA4 | *CD3D, CD3E, CD4, FOXP3, CTLA4* |
| 4.CD4T_04_CCR6 | *CD3D, CD3E, CD4, CCR6* |
| 5.CD4T_05_CCR7^lo^_GZMK | *CD3D, CD3E, CD4, CCR7, GZMK* |
| 6.CD4T_06_CD69 | *CD3D, CD3E, CD4, CD69* |
| 7.CD4T_07_IFIT3 | *CD3D, CD3E, CD4, IFIT3* |
| 8.CD8T_01_GZMK | *CD3D, CD3E, CD8A, CD8B, GZMK* |
| 9.CD8T_02_FGFBP2 | *CD3D, CD3E, CD8A, CD8B, FGFBP2* |
| 10.CD8T_03_CCR7 | *CD3D, CD3E, CD8A, CD8B, CCR7* |
| 11.CD8T_04_CCR7^lo^_GZMK | *CD3D, CD3E, CD8A, CD8B, CCR7, GZMK* |
| 12.CD8T_05_KLRB1 | *CD3D, CD3E, CD8A, CD8B, KLRB1* |
| 13.CD8T_06_STMN1 | *CD3D, CD3E, CD8A, CD8B, STMN1, MKI67* |
| 14.NKT | *CD3D, CD3E, NKG7, GNLY, KLRD1* |
| 15.NK_01_FCGR3A_CX3CR1 | *NKG7, GNLY, KLRD1, FCGR3A, CX3CR1* |
| 16.NK_02_XCL1 | *NKG7, GNLY, KLRD1, IL2RB, XCL1, XCL2* |
| 17.Mo_01_CD14 | *FCN1, S100A8, S100A9, LYZ, CD14* |
| 18.Mo_02_CD16 | *FCN1, S100A8, S100A9, LYZ, FCGR3A* |
| 19.DC_CD1C | *FCN1, S100A8, S100A9, LYZ, CD14, CD1C, CD86* |
| 20.HSPC | *CD34* |
| 21.Platelet | *ITGB3, MYL9, PPBP* |
| 22.B_01_MS4A1_CD83 | *CD79A, MS4A1, TCL1A, FCER2, CD83* |
| 23.B_02_MZB1 | *MZB1, XBP1, IGKC, JCHAIN, ITM2C* |

CD4T, CD4+ T cells; CD8T, CD8+ T cells; NKT, nature killer T cells; NK, natural killer cells; Mo, monocyte cells; DC, dendritic cells; HSPC, hematopoietic stem and progenitor cells

**Table S4. Cell numbers of the identified clusters in each sample**

| **Sample** | **P21-b** | **P21-a** | **P25-b** | **P25-a** | **P26-b** | **P26-a** | **P29-b** | **P29-a** |
| --- | --- | --- | --- | --- | --- | --- | --- | --- |
| 1.CD4T_01_CCR7 | 1,272 | 2,469 | 1,316 | 1,878 | 4,369 | 2,567 | 1,181 | 1,456 |
| 2.CD4T_02_GPR183 | 801 | 3,054 | 1,333 | 1,369 | 1,299 | 932 | 1,434 | 1,425 |
| 3.CD4T_03_FOXP3_CTLA4 | 208 | 916 | 397 | 700 | 286 | 380 | 468 | 629 |
| 4.CD4T_04_CCR6 | 178 | 759 | 467 | 502 | 231 | 228 | 512 | 525 |
| 5.CD4T_05_CCR7^lo^_GZMK | 113 | 421 | 245 | 232 | 163 | 143 | 343 | 449 |
| 6.CD4T_06_CD69 | 212 | 245 | 329 | 283 | 456 | 235 | 122 | 289 |
| 7.CD4T_07_IFIT3 | 23 | 187 | 35 | 42 | 89 | 31 | 52 | 57 |
| 8.CD8T_01_GZMK | 99 | 475 | 302 | 289 | 80 | 287 | 180 | 302 |
| 9.CD8T_02_FGFBP2 | 27 | 350 | 704 | 482 | 49 | 205 | 258 | 428 |
| 10.CD8T_03_CCR7 | 520 | 836 | 474 | 479 | 227 | 148 | 207 | 231 |
| 11.CD8T_04_CCR7^lo^_GZMK | 116 | 332 | 325 | 311 | 125 | 136 | 244 | 341 |
| 12.CD8T_05_KLRB1 | 22 | 113 | 102 | 97 | 36 | 58 | 69 | 57 |
| 13.CD8T_06_STMN1 | 2 | 435 | 6 | 95 | 3 | 122 | 9 | 107 |
| 14.NKT | 5 | 18 | 9 | 6 | 7 | 5 | 3 | 11 |
| 15.NK_01_FCGR3A_CX3CR1 | 146 | 950 | 634 | 264 | 45 | 220 | 112 | 217 |
| 16.NK_02_XCL1 | 4 | 70 | 6 | 15 | 8 | 49 | 9 | 19 |
| 17.Mo_01_CD14 | 183 | 138 | 905 | 105 | 219 | 144 | 200 | 117 |
| 18.Mo_02_CD16 | 32 | 15 | 103 | 7 | 2 | 21 | 7 | 2 |
| 19.DC_CD1C | 5 | 1 | 29 | 1 | 0 | 1 | 0 | 0 |
| 20.HSPC | 0 | 10 | 4 | 3 | 8 | 3 | 0 | 1 |
| 21.Platelet | 12 | 0 | 413 | 3 | 41 | 1 | 38 | 3 |
| 22.B_01_MS4A1_CD83 | 58 | 49 | 14 | 25 | 124 | 32 | 48 | 17 |
| 23.B_02_MZB1 | 5 | 39 | 4 | 1 | 2 | 13 | 0 | 0 |
| Total | 4,043 | 11,882 | 8,156 | 7,189 | 7,869 | 5,961 | 5,496 | 6,683 |

CD4T, CD4+ T cells; CD8T, CD8+ T cells; NKT, nature killer T cells; NK, natural killer cells; Mo, monocyte cells; DC, dendritic cells; HSPC, hematopoietic stem and progenitor cells; b, before UCB infusion; a, after UCB infusion

**Table S5. Cell proportions of the identified clusters in each sample**

| **Sample** | **P21-b** | **P21-a** | **P25-b** | **P25-a** | **P26-b** | **P26-a** | **P29-b** | **P29-a** |
| --- | --- | --- | --- | --- | --- | --- | --- | --- |
| 1.CD4T_01_CCR7 | 31.46 | 20.78 | 16.14 | 26.12 | 55.52 | 43.06 | 21.49 | 21.79 |
| 2.CD4T_02_GPR183 | 19.81 | 25.7 | 16.34 | 19.04 | 16.51 | 15.63 | 26.09 | 21.32 |
| 3.CD4T_03_FOXP3_CTLA4 | 5.14 | 7.71 | 4.87 | 9.74 | 3.63 | 6.37 | 8.52 | 9.41 |
| 4.CD4T_04_CCR6 | 4.4 | 6.39 | 5.73 | 6.98 | 2.94 | 3.82 | 9.32 | 7.86 |
| 5.CD4T_05_CCR7^lo^_GZMK | 2.79 | 3.54 | 3 | 3.23 | 2.07 | 2.4 | 6.24 | 6.72 |
| 6.CD4T_06_CD69 | 5.24 | 2.06 | 4.03 | 3.94 | 5.79 | 3.94 | 2.22 | 4.32 |
| 7.CD4T_07_IFIT3 | 0.57 | 1.57 | 0.43 | 0.58 | 1.13 | 0.52 | 0.95 | 0.85 |
| 8.CD8T_01_GZMK | 2.45 | 4 | 3.7 | 4.02 | 1.02 | 4.81 | 3.28 | 4.52 |
| 9.CD8T_02_FGFBP2 | 0.67 | 2.95 | 8.63 | 6.7 | 0.62 | 3.44 | 4.69 | 6.4 |
| 10.CD8T_03_CCR7 | 12.86 | 7.04 | 5.81 | 6.66 | 2.88 | 2.48 | 3.77 | 3.46 |
| 11.CD8T_04_CCR7^lo^_GZMK | 2.87 | 2.79 | 3.98 | 4.33 | 1.59 | 2.28 | 4.44 | 5.1 |
| 12.CD8T_05_KLRB1 | 0.54 | 0.95 | 1.25 | 1.35 | 0.46 | 0.97 | 1.26 | 0.85 |
| 13.CD8T_06_STMN1 | 0.05 | 3.66 | 0.07 | 1.32 | 0.04 | 2.05 | 0.16 | 1.6 |
| 14.NKT | 0.12 | 0.15 | 0.11 | 0.08 | 0.09 | 0.08 | 0.05 | 0.16 |
| 15.NK_01_FCGR3A_CX3CR1 | 3.61 | 8 | 7.77 | 3.67 | 0.57 | 3.69 | 2.04 | 3.25 |
| 16.NK_02_XCL1 | 0.1 | 0.59 | 0.07 | 0.21 | 0.1 | 0.82 | 0.16 | 0.28 |
| 17.Mo_01_CD14 | 4.53 | 1.16 | 11.1 | 1.46 | 2.78 | 2.42 | 3.64 | 1.75 |
| 18.Mo_02_CD16 | 0.79 | 0.13 | 1.26 | 0.1 | 0.03 | 0.35 | 0.13 | 0.03 |
| 19.DC_CD1C | 0.12 | 0.01 | 0.36 | 0.01 | 0 | 0.02 | 0 | 0 |
| 20.HSPC | 0 | 0.08 | 0.05 | 0.04 | 0.1 | 0.05 | 0 | 0.01 |
| 21.Platelet | 0.3 | 0 | 5.06 | 0.04 | 0.52 | 0.02 | 0.69 | 0.04 |
| 22.B_01_MS4A1_CD83 | 1.43 | 0.41 | 0.17 | 0.35 | 1.58 | 0.54 | 0.87 | 0.25 |
| 23.B_02_MZB1 | 0.12 | 0.33 | 0.05 | 0.01 | 0.03 | 0.22 | 0 | 0 |

CD4T, CD4+ T cells; CD8T, CD8+ T cells; NKT, nature killer T cells; NK, natural killer cells; Mo, monocyte cells; DC, dendritic cells; HSPC, hematopoietic stem and progenitor cells; b, before UCB infusion; a, after UCB infusion
