# Supplementary material for: A phase 2 pilot study of umbilical cord blood infusion as an adjuvant consolidation therapy in elderly patients with acute myeloid leukemia
Source: Signal Transduct Target Ther. 2024 Dec 20;9:358. doi: 10.1038/s41392-024-02065-y (PMC11659310; doi:10.1038/s41392-024-02065-y)
Supplement: Supplementary file 2 — Clinical Study Protocol [file 41392_2024_2065_MOESM2_ESM.docx]

**Clinical Study Protocol**

**A phase 2 pilot study of umbilical cord blood infusion as an adjuvant consolidation therapy in elderly patients with acute myeloid leukemia**

Chinese Clinical Trial Registry: ChiCTR-OPC-15006492

Ethics Approval Code: RJ-201646

Version Number : 2.0

**Ruijin Hospital affiliated to Shanghai Jiao Tong University**

**School of Medicine, Shanghai, China**

**Study statistician: Jinzeng Wang**

**Clinical** **trial investigators: Xiaoyang Li, Junmin Li**

**Translational study investigators: Jinzeng Wang, Ping Liu, Ruibao Ren**

**List of Abbreviations**

| **Abbreviations** | **Full Name** |
| --- | --- |
| AML | Acute myeloid leukemia |
| UCB | Umbilical cord blood |
| OS | Overall survival |
| EFS | Event free survival |
| MRD | Measurable residual disease |
| AE | Adverse events |
| WHO | World Health Organization |
| ECOG | Eastern Cooperative Oncology Group |
| LVEF | Left Ventricular Ejection Fraction |
| eGFR | Estimated glomerular filtration rate |
| ALT | Alanine aminotransferase |
| AST | Aspartate aminotransferase |
| ULN | Upper limit of normal |
| CR | Complete remission |
| Allo-HSCT | Allogenic hematopoietic stem cell transplantation |
| APL | Acute promyelocytic leukemia |
| sAML | secondary AML |
| CNS | Central nervous system |
| HIV | Human immunodeficiency virus |
| HBV | Hepatitis B virus |
| HCV | Hepatitis C virus |
| NYHA | New York Heart Association |
| NCI | National Cancer Institute |
| CTCAE | Common Terminology Criteria for Adverse Events |
| ICH | International Conference on Harmonization |
| GCP | Good Clinical Practice |
| CRF | Case report form |
| β2-MG | β2-microglobulin |
| AIDS | Acquired immunodeficiency syndrome |
| DAC | Decitabine |
| Ara-C | Cytarabine |
| GVHD | Graft Versus Host Disease |
| aGVHD | acute Graft Versus Host Disease |
| HLA | Human Leukocyte Antigen |
| LAIP | Leukemia-associated immunophenotyping |
| G-CSF | Granulocyte colony stimulating factor |
| GM-CSF | Granulocyte-macrophage colony stimulating factor |
| DLT | Dose-limiting toxicity |
| TPO | Thrombopoietin |
| CRA | Cancer-Related Anemia |
| EPO | Erythropoiesis-stimulating agents |
| HGB | Hemoglobin |
| HCT | Hematocrit |
| CINV | Chemotherapy-Induced Nausea and Vomiting |
| MSC | Mesenchymal Stem Cell |
| ECP | Extracorporeal Photopheresis |
| DCF | Data Clarification Form |
| FAS | Full Analysis Set |
| ITT | Intention-to-treat |
| PPS | Per-Protocol Set |
| IEC | Independent Ethics Committee |
| IRB | Institutional Review Board |
| SOPs | Standard operating procedures |

**Study Synopsis**

| **Study title** | A phase 2 pilot study of umbilical cord blood infusion as an adjuvant consolidation therapy in elderly patients with acute myeloid leukemia |
| --- | --- |
| **Indication** | AML consolidation therapy |
| **Objectives** | To assess the efficacy and safety of UCB infusion as an adjuvant consolidation therapy in elderly patients with AML |
| **Study design** | A single-arm, single-center, phase 2 trial |
| **Study center** | Ruijin Hospital affiliated to Shanghai Jiao Tong University  School of Medicine |
| **Participants** | Elderly patients (age ≥ 60 years) with newly diagnosed AML |
| **Study endpoints** | **Primary endpoint**: overall survival (OS)  **Secondary endpoints**: event-free survival (EFS), bone marrow measurable residual disease (MRD), treatment-related adverse events (AEs), and median times to platelet and neutrophil recovery  **Exploratory endpoint**: single-cell RNA sequencing (scRNA-seq) of matched samples collected before and after UCB infusion |
| **Sample size** | 65 elderly patients diagnosed newly with de novo AML were assessed for eligibility and 51 were enrolled. |
| **Inclusion criteria** | 1. Age ≥ 60 years old; 2. Newly diagnosed with de novo AML, examined by bone marrow according to the World Health Organization (WHO) 2022 criteria; 3. Eastern Cooperative Oncology Group (ECOG) performance score 0 to 2; 4. Echocardiography examination of Left Ventricular Ejection Fraction (LVEF) ≥ 50%; 5. Estimated glomerular filtration rate (eGFR) ≥ 60 mL/min, measured using the CKD-EPI formula; 6. Alanine aminotransferase (ALT) ≤ 2.5×upper limit of normal (ULN), aspartate aminotransferase (AST) ≤ 2.5×ULN and total bilirubin ≤ 1.5×ULN; 7. Documented CR after one or two cycles of induction therapies defined according to standard criteria; 8. Inability or unwillingness to undergo allo-HSCT; 9. Signed informed consent before admission to the study. |
| **Exclusion criteria** | 1. Acute promyelocytic leukemia (APL); 2. Secondary AML (sAML); 3. Subjects who had been pretreated with other investigational drugs and/or currently participate in any other clinical trials; 4. Subjects with known involvement of central nervous system (CNS); 5. Subjects with known history of human immunodeficiency virus (HIV) infection; 6. Subjects with known history of hepatitis B virus (HBV) or hepatitis C virus (HCV) infection; 7. New York Heart Association (NYHA) functional classification higher than grade 2; 8. Subjects with chronic respiratory disease requiring continuous oxygen inhalation; 9. Subjects with other malignant tumors or hematological system diseases; 10. Subjects with uncontrolled systemic infection (viral, bacterial or fungal); 11. Subjects with known or suspected autoimmune diseases; 12. Subjects with known history of intolerance or allergy to congeneric drugs; 13. Inability or unwillingness to follow the required protocol procedures; 14. Familial, psychological, geographical or sociological factors potentially impeding compliance with the protocol procedures and follow-up schedules; 15. Any uncontrolled or serious medical disorders that, at the discretion of the investigators, may increase the risks related to study participants or drug administrations, impair the abilities of the patients to undergo protocol therapies or obstruct the data interpretation of the study. |
| **Study treatment** | Two cycles of 15 mg/m2 low dose intravenous decitabine, 1.0 g/m2 intermediate-dose cytarabine, combined with one unite UCB infusion |
| **Efficacy analysis** | **Primary efficacy analysis:**  The primary endpoint of this trial was OS, defined as the time interval from the date of diagnosis to the date of death from any cause, with censoring of patients known to be alive upon the last follow-up.  **Secondary efficacy analysis:**  Secondary endpoints included EFS, bone marrow MRD by flow cytometry, and the median times to platelet and neutrophil count recovery. EFS was determined as the time interval from the date of diagnosis to the date of occurrence of any following events, including relapse or death, whichever came first.  **Exploratory efficacy analysis:**  Blood-based biological characteristics exploratory study analyzed by scRNA-seq of matched pre- and post-UCB infusion samples. |
| **Safety measures** | Treatment-related AEs, including hematological and non-hematological AEs, were defined as those that occurred from the start of treatment. Early deaths were defined as deaths occurring within 30 days of treatment with this regimen. The severity of AEs was assessed following the Common Terminology Criteria for Adverse Events (CTCAE, v5.0) of National Cancer Institute during treatment. |
| **Statistical analysis** | Clinical characteristics of patients were summarized by utilizing frequencies (%) for categorical variables and medians (range) for continuous variables. Fisher’s exact test was used to compare the associations for categorical variables versus categorical variables. Wilcoxon rank sum test was employed to compare categorical variables versus continuous variables. False discovery rate (FDR) corrected by Benjamini-Hochberg approach was applied to adjust the p value for multiple testing unless otherwise specified. Kaplan–Meier survival curves were utilized to evaluate the probabilities of OS and EFS. Log-rank test was performed to compare the survival curves. Asterisks define significance levels. All statistical tests were two-sided unless otherwise specified. Statistical analyses were carried out using R (v4.1.0) packages. |
| **Enrollment time** | 12 January 2015 to 12 February 2022 |
| **Final follow-up** | 31 March 2023 |

**Table of Contents**

1. Background

2. Study Objectives and Endpoints

2.1 Study Objectives

2.2 Study Endpoints

3. Study Design and Planned Sample Size

3.1 Study Design

3.2 Planned Sample Size

4. Participants

4.1 Inclusion Criteria

4.2 Exclusion Criteria

5. Study Procedures

5.1 Baseline Examination and Screening

5.2 Study Treatment

5.3 Evaluation and Examination During Treatment

5.4 Monitoring of Minimal Residual Disease (MRD)

5.5 Follow-up Evaluation

5.6 Concomitant Medications and Management

6. Efficacy Evaluation

6.1 Efficacy Indicators

6.2 Definitions of Efficacy Indicators

7. Safety Evaluation

7.1 Safety Observation Indicators

7.2 Definition of Adverse Events (AEs)

7.3 Serious Adverse Events (SAEs)

7.4 Common Adverse Reactions of Decitabine

7.5 Common Adverse Reactions of Cytarabine

7.6 Management of Common Adverse Reactions Related to Drugs

8. Data Management

8.1 Completion and Transfer of Case Report Forms (CRFs)

8.2 Data Entry and Modification

8.3 Data Locking

9. Data Statistics and Analysis

9.1 General Arrangement

9.2 Analysis Population

9.3 Main Results and Analysis Methods

10. Ethics

10.1 Responsibilities of the Investigator

10.2 Independent Ethics Committee (IEC)/Institutional Review Board (IRB)

10.3 Informed Consent

10.4 Confidentiality of Participant Data

11. Management Requirements

11.1 Modification of the Study Protocol

11.2 Completion of CRFs

12. Quality Control and Quality Assurance

12.1 Quality Control

12.2 Quality Assurance

12.3 Study Drug Management

13. Data Preservation and Summarization

14. Responsibilities of Parties and Publication of Papers

15. Progress of the Trial

16. References

**1. Background**

Acute myeloid leukemia (AML) is a clonal malignancy, characterized by aberrant differentiation of hematopoietic stem and progenitor cells. The global incidence of AML increased to 144,645 in 2021, with the age-standardized incidence rate of 1.73/100,000.^1^ Particularly, the incidence of AML increases with age. American and China had the highest incidence cases, with 21,533 and 17,835 AML cases in 2021, respectively.^1^ Moreover, American also showed the highest deaths while China had the highest disability-adjusted life years of AML.^1^

AML treatments consist of initial induction therapy and post-remission therapy. Despite a high initial complete remission (CR) rate after induction therapy,^2,3^ most patients die of relapsed AML. Relapse is the most frequent cause of treatment failure in AML.^4^ Moreover, older patients (age ≥ 60 years) with AML in CR have a much higher relapse rate of 80-90% compared to less than 50% in younger patients.^5,6^ Thus, appropriate post-remission therapy is critical once CR is achieved after induction therapy.

Common post-remission treatment strategies include additional cytotoxic chemotherapy (such as high or intermediate dose of cytarabine) or allogenic hematopoietic stem cell transplantation (allo-HSCT), with or without targeted therapy. Currently, allo-HSCT remains the potentially curative option in patients with AML. Most patients aged ≥ 60 years cannot proceed to allo-HSCT, due to multiple reasons including lack of donor, personal choice and biological factors. As previously reported, only 24% of older patients proceeded to allo-HSCT after intensive therapy.^7^ Meanwhile, targeted therapy may not be available due to lack of targets or lack of access for many patients. Consolidation with chemotherapy is thus administered for these patients. Unfortunately, high or intermediate dose of cytarabine consolidation did not produce prognosis benefits in elderly patients with AML.^8-10^ This leads to highly unsatisfactory outcomes in elderly AML patients. There remains an unmet medical need for these patients.

In recent years, umbilical cord blood (UCB) has emerged as a promising source with effective anti-aging roles,^11,12^ and has been studied for novel indications in malignancies.^13^ UCB has distinct practical advantages of rapid availability, easy collection, more tolerant of Human Leukocyte Antigen (HLA) mismatches, and lower risks of graft-versus-host disease (GVHD).^14^ Given its accessibility, UCB can be available for most of the patients with AML in need. Based on these, we designed a prospective, single-arm, phase 2 clinical trial to investigate the efficacy and safety of consolidation regimen of decitabine and cytarabine combined with UCB infusion in elderly AML patients.

**2. Study Objectives and Endpoints**

2.1 Study Objectives

This study was aimed to evaluate the efficacy and safety of decitabine, cytarabine combined with unrelated UCB infusion as a consolidation therapy in elderly patients with AML after achieving the first CR (CR1).

2.2 Study Endpoints

2.2.1 Primary Endpoint

- Overall Survival (OS): defined as the time interval from the date of diagnosis to date of death from any cause, with censoring of patients known to be alive upon the last follow-up.

2.2.2 Secondary Endpoints

- Event-free Survival (EFS): defined as the time interval from the date of diagnosis to the date of occurrence of any following events, including relapse or death, whichever came first.

- Bone marrow measurable residual disease (MRD) by flow cytometry.

- The median times to recovery of neutrophils (≥ 0.5 × 10⁹/L) and platelets (≥ 20 × 10⁹/L and ≥ 50 × 10⁹/L) documented for each patient.

- Safety: including hematological and non-hematological adverse events (AEs) and the severity of AEs assessed following the National Cancer Institute Common Terminology Criteria for Adverse Events (NCI-CTCAE, v5.0).

2.2.3 Exploratory Endpoint

- Single-cell RNA sequencing (scRNA-seq) of matched samples collected before and after UCB infusion.

**3. Study Design and Planned Sample Size**

3.1 Study Design

This was a prospective, single-arm, open label, phase 2 study evaluating UCB infusion as an adjuvant consolidation regimen in elderly AML patients (ChiCTR-OPC-15006492). This trial was approved by the Human Ethics Committee of Ruijin Hospital affiliated to Shanghai Jiao Tong University School of Medicine (RJ-201646). The protocol was in accordance with Declaration of Helsinki Principles and International Conference on Harmonization (ICH) Good Clinical Practice (GCP) Guidelines. After the institutional review board approval, this trial recruited patients at Ruijin Hospital.

3.2 Planned Sample Size

This study is a phase 2 clinical trial using a single-arm, single-stage design method to calculate the sample size. The primary endpoint is the 2-year OS of all enrolled patients. Based on prior knowledge, the 2-year OS with standard chemotherapy is about 50%. Assuming the 2-year OS expectation with UCB treatment is 67%, with P1 = 0.67 (superior) and P0 = 0.50 (inferior), α = 0.05, β = 0.20, and considering a 5% expected dropout rate, the study plans to enroll approximately 52 patients.

**4. Participants**

4.1 Inclusion Criteria:

1. Age ≥ 60 years old;
2. Newly diagnosed with de novo AML, examined by bone marrow according to the World Health Organization (WHO) 2022 criteria;
3. Eastern Cooperative Oncology Group (ECOG) performance score 0 to 2;
4. Echocardiography examination of Left Ventricular Ejection Fraction (LVEF) ≥ 50%;
5. Estimated glomerular filtration rate (eGFR) ≥ 60 mL/min, measured using the CKD-EPI formula;
6. Alanine aminotransferase (ALT) ≤ 2.5×upper limit of normal (ULN), aspartate aminotransferase (AST) ≤ 2.5×ULN and total bilirubin ≤ 1.5×ULN;
7. Documented CR after one or two cycles of induction therapies defined according to standard criteria;
8. Inability or unwillingness to undergo allo-HSCT;
9. Signed informed consent before admission to the study.

4.2 Exclusion Criteria:

1. Acute promyelocytic leukemia (APL);
2. Secondary AML (sAML);
3. Subjects who had been pretreated with other investigational drugs and/or currently participate in any other clinical trials;
4. Subjects with known involvement of central nervous system (CNS);
5. Subjects with known history of human immunodeficiency virus (HIV) infection;
6. Subjects with known history of hepatitis B virus (HBV) or hepatitis C virus (HCV) infection;
7. New York Heart Association (NYHA) functional classification higher than grade 2;
8. Subjects with chronic respiratory disease requiring continuous oxygen inhalation;
9. Subjects with other malignant tumors or hematological system diseases;
10. Subjects with uncontrolled systemic infection (viral, bacterial or fungal);
11. Subjects with known or suspected autoimmune diseases;
12. Subjects with known history of intolerance or allergy to congeneric drugs;
13. Inability or unwillingness to follow the required protocol procedures;
14. Familial, psychological, geographical or sociological factors potentially impeding compliance with the protocol procedures and follow-up schedules;
15. Any uncontrolled or serious medical disorders that, at the discretion of the investigators, may increase the risks related to study participants or drug administrations, impair the abilities of the patients to undergo protocol therapies or obstruct the data interpretation of the study.

**5. Study Procedures**

5.1 Baseline Examination and Screening

1. Medical History Inquiry: Before the start of the study treatment, demographic characteristics including age, sex, smoking history, cancer history, other medical histories (active or cured), comorbidities, and concomitant medications were collected according to the protocol and recorded in the case report form (CRF).
2. Pre-Treatment Baseline Examination:

- To be conducted within 7 days before the start of the study medication.

- Vital signs, physical examination, ECOG score.

- Complete blood count, biochemical routine, coagulation function, EBV-DNA, serum amylase, urine protein and urinalysis, stool routine + occult blood, serum β2-microglobulin (β2-MG).

- Six items of cardiac function, thyroid function, blood type identification, infection screening (hepatitis B, hepatitis C, AIDS, syphilis; if abnormal, complete viral load such as HBV-DNA, HCV-RNA detection).

- Echocardiography, abdominal ultrasound scan, chest CT scan, and electrocardiogram.

- Bone marrow examination: bone marrow smear + flow cytometry MRD detection.

1. Patients who meet the inclusion criteria and do not meet the exclusion criterial were enrolled in the study.

5.2 Study Treatment

Consolidation Therapy:

- Two cycles of 15 mg/m^2^ low dose intravenous decitabine (DAC) over 4h per day for consecutive 5 days (day 1-5), 1.0 g/m^2^ intermediate-dose cytarabine (Ara-C) at q12h for consecutive 2 days (day 6-7), combined with one unit UCB infusion on day 9.

- No immunosuppression was given as prophylaxis for GVHD, unless acute GVHD (aGVHD) was documented or clinically diagnosed.

Selection of UCB:

- High-resolution Human Leukocyte Antigen (HLA) typing for HLA-A, B, and DR loci was performed in all enrolled patients.

- The UCB units were obtained from the cord blood bank at the China Shandong Cord Blood Bank Network if they met the following criteria:

a). Serologically matched of four or five of six HLAs.

b). Contained at least 3 × 10⁷ nucleated cells/kg of recipient body weight before freezing.

Maintenance Therapy:

- Decitabine 15 mg/m² for 5 days, every 28-day cycle.

OR

- Azacitidine 75 mg/m² for 7 days, every 28-day cycle.

5.3 Evaluation and Examination During Treatment

1. After completing each treatment cycle, patients were required to have a complete blood count examination every 3 days and coagulation function, liver, and kidney function examinations weekly. The main indicator values were documented during the process.
2. Physical examination, toxicity assessment, and pre-chemotherapy assessment (including complete blood count, biochemical routine, coagulation function, EBV-DNA, serum amylase, urine protein and urinalysis, stool routine + occult blood, serum β2-MG, thyroid function, six items of cardiac function, electrocardiogram, echocardiography, and for hepatitis B or C patients, HBV-DNA and HCV-RNA testing).
3. Chimerism Analysis: on the day 7 after UCB infusion, peripheral blood cells were collected from all patients and assessed for chimerism using standard cytogenetic and a semi-quantitative polymerase chain reaction based assay of short tandem repeats with the sensitivity of 1%.

5.4 Monitoring of Minimal Residual Disease (MRD)

- Leukemia-associated immunophenotyping (LAIP) was determined at diagnosis using different surface antigens.

- Monoclonal antibodies against 20 antigens were utilized: CD2, cyCD3, CD4, CD7, CD11b, CD13, CD14, CD15, CD19, CD33, CD34, CD38, CD45, CD56, CD64, cyCD79a, CD117, HLA-DR, MPO, and TdT.

- A cut-off value of 0.01% was set for defining MRD negativity.

5.5 Follow-up Evaluation

Schedules:

- Post-UCB treatment on days 14, 30, and 60.

- Before each chemotherapy session and before and after each maintenance treatment during the treatment period.

- After the end of treatment, follow-up assessments were performed monthly in the first year, and every three months thereafter.

Testing Items:

- Bone marrow aspiration smear, MRD, bone marrow chimerism detection.

- Chromosome testing was required after consolidation and maintenance therapy.

- If there was any disease recurrence detected by bone marrow cytology at any time during the treatment, the treatment will be deemed ineffective. Then the patient will be withdrawn from this study and enter the trials for relapsed and refractory AML treatment.

- If bone marrow chimerism > 30% at any time during treatment, donor engraftment will be considered, and the patient will be withdrawn from the clinical trial and receive anti-rejection treatment.

5.6 Concomitant Medications and Management

5.6.1 Permitted Concomitant Treatments:

- Antiemetics may be used prior to the administration of chemotherapy drugs.

- Blood products (suspended red blood cells, single donor platelets, plasma, Fg, etc.) may be used as supportive therapy.

- During the treatment process, broad-spectrum antibiotics (antibacterial/antifungal), growth factors (G-CSF/GM-CSF, IL-11, and TPO, etc.), and hepatoprotective drugs may be used, according to the patient's condition.

5.6.2 Prohibited Treatments:

- During the trial, patients were prohibited from using other experimental drugs, radiotherapy, chemotherapy, and other treatments that may interfere with the study.

**6. Efficacy Evaluation**

6.1 Efficacy Indicators

- OS

- EFS

- Neutrophil and platelet recovery

6.2 Definitions of Efficacy Indicators

- OS: defined as the time interval from the date of diagnosis to the date of death from any cause, with censoring of patients known to be alive upon the last follow-up.

- EFS: determined as the time interval from the date of diagnosis to the date of occurrence of any following events, including relapse or death, whichever came first.

- Neutrophil and platelet recovery: the median times to recovery of neutrophils (≥ 0.5 × 10⁹/L) and platelets (≥ 20 × 10⁹/L and ≥ 50 × 10⁹/L).

**7. Safety Evaluation**

The investigator is responsible for monitoring the safety of all enrolled subjects and recording any adverse events (AEs) and serious adverse events (SAEs). AEs monitoring began after the first patient enrollment and was monitored continuously until the last follow-up.

7.1 Safety Observation Indicators

- Medical History Inquiry: Comprehensive recording of clinical characteristics and causes of patient complaints.

- Physical Examination: Height, weight, vital signs (including ECOG score), and examination of various organs.

- Laboratory Examination Indicators: Complete blood count, biochemical routine, urinalysis, stool routine, EBV-DNA, coagulation function, thyroid function, six items of cardiac function, NYHA cardiac function classification, and 12-lead electrocardiogram.

7.2 Definition of Adverse Events (AEs)

Treatment-related AEs, including hematological and non-hematological AEs, were defined as those that occurred from the start of treatment. For pre-existing abnormalities in physical or laboratory examinations, an increase in severity after treatment was also considered as an AE. The investigator should record all AEs in detail, including descriptions and related symptoms, time of occurrence, severity, duration, measures taken, and outcomes.

7.2.1 Criteria for Determining the Severity of AEs

The severity of AEs was graded according to the NCI-CTCAE v5.0 criteria. For adverse reactions not listed in the table, refer to the following standards:

- Grade I: Mild, no clinical symptoms or mild clinical symptoms; only clinical or laboratory examination abnormalities; no treatment required.

- Grade II: Moderate, minimal, local, or noninvasive treatment required; age-appropriate tools indicate limited daily activities, including cooking, shopping, using the phone, and handling money.

- Grade III: Severe disease or significant clinical symptoms but not life-threatening; requires hospitalization or prolongs hospitalization; causes disability; limited self-care daily activities (e.g., bathing, dressing, eating, using the toilet, taking medication, not bedridden).

- Grade IV: Life-threatening, requiring emergency treatment.

- Grade V: Death due to AE.

7.2.2 Criteria for Determining the Relationship Between AEs and Study Drug

All AEs must be reported in clinical reports, and any discomfort or abnormal changes in objective laboratory indicators during the trial must be recorded accurately, indicating the severity, duration, treatment measures, and outcomes. Clinicians should use a five-category method ("definitely related," "probably related," "possibly unrelated," "definitely unrelated," and "unable to determine") to comprehensively judge the relationship between AE and the study drug. "Definitely related," "probably related," and "unable to determine" are classified as drug adverse reactions. The incidence of AEs is calculated as the sum of these three categories as the numerator and the total number of subjects evaluated for safety as the denominator.

| **Category** | **Criteria** |
| --- | --- |
| Definitely related | The event occurs in a reasonable sequence after drug administration, matches the known reaction type of the suspected drug, improves after discontinuation, and recurs after re-administration. |
| Probably related | The event occurs in a reasonable sequence after drug administration but does not match the known reaction type of the suspected drug; the patient's clinical status or other treatments may also cause the event. |
| Possibly unrelated | The event does not occur in a reasonable sequence after drug administration, does not match the known reaction type of the suspected drug, and the patient's clinical status or other treatments may cause the event. |
| Definitely unrelated | The event does not occur in a reasonable sequence after drug administration, does not match the known reaction type of the suspected drug, the patient's clinical status or other treatments may cause the event, and the event disappears after discontinuing other treatments or improving the disease and reappears after reintroducing other treatments. |
| Unable to determine | The event's occurrence and timing have no clear relationship with drug administration, similar to the drug reaction type, and other concurrent medications may also cause the event. |

7.3 Serious Adverse Events (SAEs)

7.3.1 Definition of SAEs

SAEs refer to medical events during a clinical trial that require hospitalization or prolong hospitalization, cause disability, affect work ability, are life-threatening or result in death, or cause congenital malformations. This includes the following unexpected medical events:

- Events leading to death.

- Life-threatening events (defined as events where the subject is at risk of death at the time of occurrence).

- Events causing significant or permanent physical disability or organ function impairment.

- Events potentially leading to permanent or serious disability/loss of work ability.

- Carcinogenic, teratogenic, or birth defects.

- Other significant medical events requiring intervention to prevent permanent damage or injury.

7.3.2 Disease Progression

Disease progression is defined as the worsening of clinical symptoms/signs of the disease for the study drug, the emergence of new metastases relative to the primary malignance, or the progression of existing lesions. Symptoms and signs caused by disease progression leading to death, life-threatening conditions, hospitalization, or extended hospitalization, causing permanent or serious disability/loss of work ability, or birth defects are not reported as SAEs. Death due to disease progression symptoms and signs should be reported as SAEs.

7.3.3 Other Antitumor Treatments

If the subject begins other antitumor treatments, non-fatal AEs should be reported until the start of new antitumor treatment. If death occurs after the end of study treatment but within the SAE reporting period, it must be reported promptly, regardless of whether the patient received other treatments.

7.3.4 Hospitalization

AEs leading to hospitalization or extended hospitalization during the clinical study should be considered SAEs, excluding hospitalization for non-medical purposes. Hospitalizations or extended hospitalizations unrelated to AE worsening are not considered SAEs. For example:

- Hospitalization for pre-existing conditions without new AE or worsening of existing conditions (e.g., laboratory abnormalities present before the trial).

- Hospitalization for administrative reasons (e.g., annual routine physical examinations).

- Hospitalization required by the clinical trial protocol (e.g., procedures required by the trial protocol).

- Elective hospitalization unrelated to AE worsening (e.g., elective cosmetic surgery).

- Pre-planned treatments or surgeries should be recorded in the overall trial protocol and/or each subject's baseline data.

- Hospitalization for blood product use only.

Diagnostic or therapeutic invasive procedures (e.g., surgery), non-invasive procedures should not be reported as AEs. However, if the condition leading to the procedure meets the definition of AE, it should be reported. For example, acute appendicitis occurring within the AE reporting period should be reported as an AE. Appendectomy performed as a result should be recorded as the treatment method for the AE.

7.3.5 SAE Reporting

During the trial, if an SAE occurs, whether it is an initial report or a follow-up report, the investigator must immediately complete the "Clinical Research SAE Report Form", sign, and date it, and report it to the Ethics Committee of the research institution. During the extended supply of the study drug after the study ends, any SAE must be reported within 24 hours after the investigator becoming aware of the SAE. All SAE information should be recorded in the SAE form. SAEs occurring within 90 days after the last administration must be reported. SAEs occurring after 90 days post-administration should generally not be reported unless they are suspected to be related to the study drug. SAEs should be recorded in detail, including symptoms, severity, time of occurrence, treatment time, measures taken, follow-up time and method, and outcome. If the investigator believes the SAE is unrelated to the study drug but potentially related to study conditions (e.g., discontinuation of the original treatment protocol or complications during the trial), the specific relationship should be described in the case report form's SAE page. If the intensity of the ongoing SAE or its relationship to the study drug changes, an SAE follow-up report should be sent to the investigator immediately. All SAEs should be followed up until recovery or stabilization of the condition.

Serious Adverse Event Contact Information

| **Unit** | **Contact Department** | **Reporting Method** | **Contact Information** |
| --- | --- | --- | --- |
| Ruijin Hospital | Ethics Committee | In-person | Phone: +86-021-64370045 |

7.3.6 AEs of Special Interest

If any of the following AEs occur during the clinical trial, they must be reported to the investigator within 24 hours, even if they do not meet the SAE definition. If they also qualify as SAEs, the "Serious Adverse Event Report Form" must also be completed.

1. Infusion reactions of grade ≥ 3 (even if not dose-limiting toxicity, DLT).
2. Diarrhea/colitis, uveitis, or interstitial pneumonia of grade ≥ 2.
3. Other immune-related AEs of grade ≥ 3.
4. Any potential Hy's Law event (ALT/AST > 3× ULN, TBIL > 2× ULN without other related causes).
5. Any grade of GVHD.

7.3.7 Infusion Reactions

For potential infusion and/or allergic reactions, especially acute infusion reactions, the investigator needs to closely monitor throughout the study. Generally, no premedication is required before UCB infusion. According to published data,^15^ immediate-type allergic reactions/anaphylaxis are most likely to occur within 24 hours after infusion. If they occur, the infusion should be slowed or interrupted, clinical supportive treatment should be given, and premedication should be administered before the next infusion. Possible allergic reactions include fever, chills, tremors, headache, rash, itching, arthralgia, hypotension or hypertension, or bronchospasm. All grade 3 or 4 infusion reactions, whether they meet DLT criteria or not, should be reported according to SAE procedures. The management of allergic reactions should follow the medical practices and guidelines of the research institution. The following are suggested guidelines for managing infusion reactions.

7.3.8 Follow-up of AEs/SAEs

All SAEs and drug-related AEs should be followed up until they disappear, return to baseline level or ≤ grade 1, the condition stabilizes, or an explanation is obtained (e.g., loss to follow-up, death). At each follow-up, the investigator should inquire about any AE/SAE occurrences since the last follow-up, and whether any new AE/SAE has occurred, recording relevant updated information, including outcomes.

7.4 Common Adverse Reactions of Decitabine

1) Hematologic Toxicity:

- Neutropenia (low neutrophil count)

- Thrombocytopenia (low platelet count)

- Anemia (low red blood cell count)

- Leukopenia (low white blood cell count)

- Febrile neutropenia (fever with low neutrophil count)

2) Infections:

- Increased susceptibility to infections, including pneumonia, sepsis, and upper respiratory infections

3) Gastrointestinal:

- Nausea

- Vomiting

- Diarrhea

- Constipation

- Abdominal pain

4) General:

- Fatigue

- Fever

- Weakness

- Edema (swelling)

5) Hepatic:

- Elevated liver enzymes (ALT, AST)

- Hyperbilirubinemia (elevated bilirubin levels)

6) Cardiovascular:

- Hypotension (low blood pressure)

7) Dermatologic:

- Rash

- Petechiae (small red or purple spots on the body)

8) Respiratory:

- Cough

- Dyspnea (difficulty breathing)

9) Musculoskeletal:

- Arthralgia (joint pain)

- Myalgia (muscle pain)

10) Renal:

- Elevated serum creatinine

11) Neurologic:

- Headache

- Dizziness

7.5 Common Adverse Reactions of Cytarabine

1) Hematologic Toxicity:

- Neutropenia (low neutrophil count)

- Thrombocytopenia (low platelet count)

- Anemia (low red blood cell count)

- Leukopenia (low white blood cell count)

2) Gastrointestinal:

- Nausea

- Vomiting

- Diarrhea

- Stomatitis (inflammation of the mouth)

- Esophagitis (inflammation of the esophagus)

- Anorexia (loss of appetite)

3) General:

- Fatigue

- Fever

- Malaise (general discomfort)

4) Hepatic:

- Elevated liver enzymes (ALT, AST)

- Hyperbilirubinemia (elevated bilirubin levels)

- Hepatotoxicity

5) Dermatologic:

- Rash

- Erythema (redness of the skin)

- Alopecia (hair loss)

- Dermatitis (inflammation of the skin)

6) Neurologic:

- Cerebellar toxicity (manifesting as ataxia, nystagmus, dysarthria)

- Peripheral neuropathy (nerve damage causing numbness or tingling)

- Headache

- Dizziness

7) Respiratory:

- Pneumonitis (inflammation of the lung tissue)

- Pulmonary edema (fluid in the lungs)

- Dyspnea (difficulty breathing)

8) Ocular:

- Conjunctivitis (inflammation of the eye)

- Keratitis (inflammation of the cornea)

9) Renal:

- Elevated serum creatinine

- Hematuria (blood in the urine)

10) Infections:

- Increased risk of bacterial, fungal, and viral infections due to immunosuppression

11) Other:

- Pain at the injection site

- Localized tissue necrosis (if extravasation occurs)

7.6 Management of Common Adverse Reactions Related to Drugs

7.6.1 Bone Marrow Suppression

1) Leukopenia

- Indications for G-CSF in the treatment of febrile neutropenia:

Sepsis, age > 65, severe neutropenia (absolute neutrophil count < 100/mcl), expected duration of neutropenia exceeding 10 days, pneumonia or other clinically documented infections, invasive fungal infections, hospitalization due to fever, history of febrile neutropenia. Some leukemia chemotherapy regimens require prophylactic G-CSF to ensure the smooth administration of chemotherapy doses and schedules. The use of prophylactic G-CSF is decided by the investigator based on the patient's actual condition.

- G-CSF Administration Method:

Pegylated G-CSF: Therapeutic or prophylactic use: Single use per cycle. Dosage: 6 mg for body weight > 45 kg; 3 mg for body weight ≤ 45 kg, administered the day after chemotherapy ends.

- Management of Febrile Neutropenia:

a). Assess infection risk and resistance, and initiate empirical antibiotics immediately.

b). Antibacterial treatment should continue throughout the neutropenia period until ANC > 0.5×10⁹/L.

c). Initial empirical antibiotic therapy aims to reduce serious complications and mortality from bacterial infections. Accurate microbiological culture results are essential.

d). Choice of empirical antibiotic therapy should consider the patient's comprehensive evaluation (risk stratification, infection site, organ function, resistance risk factors), bacteria (local and institutional epidemiology and resistance surveillance data), and the antibiotic itself (broad spectrum, pharmacokinetics/pharmacodynamics, side effects). Attention should be paid to overlapping side effects with drugs used to treat the primary disease (e.g., hematologic tumor chemotherapy drugs, immunosuppressants).

e). Low-risk patients: Initial treatment can be outpatient with the options of oral or intravenous empirical antibiotics. Combined therapy with oral ciprofloxacin, amoxicillin/clavulanate, levofloxacin, or moxifloxacin is recommended.

f). High-risk patients: Immediate hospitalization. For patients with mild conditions, a progressive strategy is taken, initially using broad-spectrum antibiotics such as cephalosporins to reduce increased bacterial resistance due to antibiotic misuse; for patients with severe conditions, a regressive strategy is taken to improve prognosis.

2) Thrombocytopenia

- Chemotherapy-Induced Thrombocytopenia (CIT): Refers to the inhibition of bone marrow, especially megakaryocytes, by chemotherapy drugs, causing peripheral blood platelet counts to drop below 100×10⁹/L.

a). Treatment of CIT: Platelet transfusion, administration of platelet growth factors such as recombinant human interleukin-11 (rhIL-11) or recombinant human thrombopoietin (TPO).

b). rhIL-11 Administration: rhIL-11 should be used when platelet counts are 25~75×10⁹/L. Recommended dose is 25~50 µg/kg, subcutaneously, once daily for at least 7-10 days. Discontinue when platelet count reaches 75×10⁹/L.

c). rhTPO Administration: If chemotherapy drugs are expected to cause thrombocytopenia and induce bleeding, requiring increased platelet counts, administer subcutaneously 6-24 hours after chemotherapy ends, at a dose of 300 U/kg daily for 7-10 days. Regularly monitor complete blood counts, usually every other day, and discontinue when platelet count reaches 75×10⁹/L.

3) Anemia

- Cancer-Related Anemia (CRA): Refers to anemia occurring during cancer progression and treatment. Laboratory tests are needed to determine the cause. Treatment principles are as follows:

a). Non-Chemotherapy-Related Anemia: Provide targeted treatment for different causes.

Nutritional anemia: Supplement with iron, folic acid, and vitamin B12; autoimmune anemia: Treat with corticosteroids (if not contraindicated); tumor-related inflammation or chemotherapy-induced bone marrow suppression: Evaluate the risk-benefit of treatment and decide whether to use blood transfusions or erythropoiesis-stimulating agents (EPO).

b). Red Blood Cell Transfusion: Quickly increases hemoglobin (HGB) and hematocrit (HCT). In China, blood transfusion is recommended when HGB < 6 g/dL. Consensus on transfusion in China: Severe or above anemia patients; moderate anemia with severe symptoms; EPO ineffective patients.

c). EPO: Takes weeks to stimulate HGB elevation but is effective in maintaining HGB at an appropriate target level. For leukemia chemotherapy-induced anemia, consider using EPO after evaluating the risk of thrombosis.

d). Parenteral Iron Supplements: Treat iron-deficiency anemia (transferrin saturation < 15%, serum ferritin < 30 ng/mL) with oral or intravenous iron supplements without simultaneous EPO. Low-molecular-weight dextran iron, ferric gluconate, or ferric sucrose are recommended due to fewer side effects.

7.6.2 Gastrointestinal Reactions

Chemotherapy-Induced Nausea and Vomiting (CINV): Common adverse reactions during cancer treatment, significantly affecting patients' quality of life and potentially leading to reduced or discontinued chemotherapy regimens.

- Anticancer drugs can be classified into high, moderate, low, and minimal emetogenic risk levels, meaning the vomiting incidence without prophylactic treatment is > 90%, 30%-90%, 10%-30%, and < 10%, respectively.

- Prophylactic Antiemetics: This protocol recommends combined use of neurokinin-1 (NK-1) receptor antagonists (aprepitant), 5-HT3 receptor antagonists, etc., before chemotherapy.

- Delayed Antiemesis: For severe vomiting after chemotherapy, administer metoclopramide 20 mg intramuscularly twice daily, oral 5-HT3 receptor antagonists (e.g., ondansetron orally disintegrating tablets), or oral NK-1 receptor antagonists (e.g., aprepitant) post-chemotherapy for antiemesis.

7.6.3 GVHD

Routine monitoring of peripheral blood microchimerism is performed after treatment, and if the chimerism rate exceeds 5%, GVHD treatment should be initiated immediately.

1). First-line Treatment:

- Corticosteroids: Methylprednisolone 1-2 mg/kg/day or equivalent dose for 2-3 weeks.

- Calcineurin Inhibitors: Such as cyclosporine A and tacrolimus.

2). Second-line Treatment (for refractory or dependent cases):

- Antithymocyte Globulin: Such as rabbit antithymocyte globulin (ATG).

- Monoclonal Antibodies: Such as anti-CD25, anti-CD52, and anti-TNF-α.

- mTOR Inhibitors: Such as sirolimus.

- Mesenchymal Stem Cell (MSC) Infusion.

- Ruxolitinib: A JAK1/2 inhibitor.

- Extracorporeal Photopheresis (ECP).

3). Supportive Treatment:

- Skin: Maintain cleanliness and prevent infection.

- Liver: Hepatoprotective treatment, discontinue hepatotoxic drugs if necessary.

- Gastrointestinal Tract: Correct electrolyte imbalances, monitor for bleeding, and withhold food if necessary.

4). Close monitoring of organ function changes, prevention and treatment of infections, and other complications. Transfer to the bone marrow transplant unit or ICU as needed.

**8. Data Management**

8.1 Completion and Transfer of Case Report Forms (CRFs)

- CRFs are to be completed by the physician, and a complete CRF must be filled out for each enrolled case. After completion and review by the investigator, the first copy of the CRF will be transferred to the data manager for data entry and management.

8.2 Data Entry and Modification

- Data entry and management are handled by data managers authorized by Ruijin Hospital, affiliated to Shanghai Jiao Tong University School of Medicine. Data managers will use Epidata software to develop the data entry program for data entry and management. To ensure data accuracy, two data entry personnel will independently perform double data entry and cross-checking.

- For issues identified in the CRFs, the data manager will generate a Data Clarification Form (DCF) and send inquiries to the investigator. The investigator should respond and return the DCF as soon as possible. The data manager will then make, confirm, and update the data modifications based on the investigator’s responses.

8.3 Data Locking

- After all inquiries have been resolved and the database is confirmed to be correct, the data manager will write a data management audit report. In the presence of the principal investigator, statistical analysts, and data management personnel, the analysis dataset will be finalized and reviewed. The locked data is considered archived data, and in principle, no further changes will be made to the archived data files.

**9. Data Statistics and Analysis**

9.1 General Arrangement

- Descriptive statistical analysis will be primarily used in this study, and statistical processing will be conducted using R (v4.1.0) software.

9.2 Analysis Population

- The analysis population includes the full analysis set, the per-protocol set, and the safety analysis set. The total number of enrolled patients, the number in each set, and the number excluded will be recorded, and the dropout cases will be described.

- Full Analysis Set (FAS): Based on the intention-to-treat (ITT) principle, the FAS includes all enrolled patients who have used the study drug at least once, excluding a minimal number of patients who have not used the study drug or have seriously violated the inclusion/exclusion criteria. Efficacy analysis will be performed on all cases that were enrolled and used the drug at least once.

- Per-Protocol Set (PPS): Based on the FAS, the PPS further excludes any protocol-violating patients, including all cases that completed at least 2 cycles (including 2 cycles), complied with the trial protocol, had good medication compliance, did not use prohibited medications during the trial, and completed the contents specified in the CRF. Missing values will not be estimated. Both FAS and PPS will be used for statistical analysis of drug efficacy.

- Safety Analysis Set: Excludes a minimal number of patients who have not used the study drug or have no safety evaluation data from the ITT set. It includes all patients who used the study drug at least once after enrollment and have safety records post-medication. This dataset is used for safety analysis.

9.3 Main Results and Analysis Methods

Clinicopathological characteristics of patients were summarized by utilizing frequencies (%) for categorical variables and medians (range) for continuous variables. Fisher’s exact test was used to compare the associations for categorical variables versus categorical variables. Wilcoxon rank sum test was employed to compare categorical variables versus continuous variables. False discovery rate (FDR) corrected by Benjamini-Hochberg approach was applied to adjust the p value for multiple testing unless otherwise specified. Kaplan–Meier survival curves were utilized to evaluate the probabilities of OS and EFS. Log-rank test was performed to compare the survival curves. Asterisks define significance levels. All statistical tests were two-sided unless otherwise specified. Statistical analyses were carried out using R (v4.1.0) packages.

**10. Ethics**

10.1 Responsibilities of the Investigator

- Investigators are responsible for ensuring that the clinical research is conducted in accordance with the trial protocol, current ICH-GCP, and relevant regulations of the National Medical Products Administration (NMPA).

10.2 Independent Ethics Committee (IEC)/Institutional Review Board (IRB)

- Before the study begins, investigators must provide the following documents to the IEC:

a). Final draft of the trial protocol (and its supplements).

b). Informed consent form and other written materials provided to participants agreed upon with the investigator.

c). Drug instructions.

d). Materials to assist in selecting participants.

e). Other documents required by the IEC.

- The trial can only begin after the IEC has fully approved the study protocol, informed consent form, and materials for selecting participants, and the investigator has received a copy of the IEC's approval document. The approval letter must specify the approved study title (number), document names (including version numbers), and approval date.

- During the trial, the investigator may need to submit the following documents to the IEC for approval due to certain reasons at appropriate times:

a). Supplementary documents to the protocol.

b). Modifications to the informed consent form and any other written materials provided to participants.

c). Revised information on compensation for injuries or participant remuneration related to the trial.

d). Supplements or updates to the drug instructions.

e). New information that may adversely affect participant safety and the implementation of the study.

f). Deviations and changes to the study protocol to avoid direct harm to participants.

g). Reports of participant deaths.

h). Notification of changes to the principal investigator at the research unit.

i). Other requirements of the IEC.

- When the supplementary protocol increases participant risk, the supplementary protocol and the corresponding revised informed consent form must be submitted to the IEC for review and approval before implementation.

10.3 Informed Consent

- Participants (or their legal representative) must provide written consent after fully understanding the study's purpose and content. This written consent must be signed (with the date) before any trial-related procedures are performed. The informed consent form should comply with the Declaration of Helsinki, current GCP guidelines, and relevant regulations.

- Before enrolling potential participants, the investigator or authorized personnel should explain the study's purpose, methods, possible benefits, risks, and any potential discomforts. Participants should be informed that participation in the trial is voluntary, and they can withdraw at any time. Whether or not they choose to participate will not affect their treatment for the disease. If a participant refuses to join the study, they can still choose other treatment options without affecting future treatment. Finally, participants should be aware that the investigator may need to retain their identity records for long-term follow-up and that their records may be reviewed by regulatory authorities and researchers within the scope of relevant laws and regulations. Participant privacy will be protected. After signing the informed consent form, participants authorize the aforementioned actions.

- Participants (or their legal representatives) should have sufficient time to read the informed consent form and ask questions. After the investigator's explanation and before enrollment, participants (or their legal representatives) should sign and date the informed consent form for archival purposes. After signing, participants should receive a copy of the informed consent form.

- If a participant (or his/her legal representative) cannot read or write, an impartial witness should participate in the entire informed consent process (including reading and explaining all written materials). After verbal agreement, the participant (or his/her legal representative) should sign and date the form on the spot.

- Participants unable to understand the informed consent content can only join the trial after their legal representative has obtained informed consent.

- If it is impossible to obtain the participant's consent in advance and the legal representative is not present, the procedure for including these participants should be clearly described in the protocol, recording the IEC's agreement and recommendations for protecting the participants' rights and health. Additionally, the legal representative should be notified and consent obtained as soon as possible.

10.4 Confidentiality of Participant Data

- The confidentiality of participant data will be strictly maintained by the investigator and research staff. The study protocol, documents, data, and all related information will be strictly confidential. Any relevant study or data information must not be disclosed to any unauthorized third party without prior written approval from the research unit. The investigator, other authorized representatives of the IRB, or representatives of the pharmaceutical company providing the study drug may inspect all documents and records that the investigator needs to retain, including but not limited to: medical records and participant medication records. The research center should allow access to these records. Participant contact information will be securely stored at the research center and used internally during the study. Participant research data collected for statistical analysis and scientific reporting will be uploaded and stored at Ruijin Hospital, affiliated to Shanghai Jiao Tong University School of Medicine. This should not include participant contact or identity information. At the end of the study, all research databases will be de-identified and archived at Ruijin Hospital.

**11. Management Requirements**

11.1 Modification of the Study Protocol

- Any modifications affecting the execution of the study, patient benefits, study objectives, study design, number of patients, and study procedures must be submitted by the investigator as amendments to the study protocol. These amendments must receive approval from the Ethics Committee before implementation.

11.2 Completion of CRFs

- CRFs must be filled out using blue or black ink pens. Required information must be entered in the mandatory fields on the CRF. If information is not applicable, enter "NA"; if a test was not performed, enter "ND"; if the information is unavailable, enter "NAV"; blank fields are not allowed. In principle, items should not be modified arbitrarily; if corrections are needed, strike through the error with a single line, write the correct information next to it, and note the date of the correction and the initials of the person making the correction. Patient names should not be written on the CRF; instead, use the patient's initials and identification number, and ensure the investigator signs and dates the CRF.

- The investigator will first determine eligible subjects based on the "Screening Form" content, then complete the CRF "Cover Page" and "Enrollment Form," summarizing the general condition of the subjects to determine eligibility. Post-enrollment, treatments and examinations will be conducted as planned, and relevant content will be recorded in the original medical records, which will then be transferred to the CRF. After the study, the form-filling physician/nurse and supervising physician will carefully review the CRF for consistency with the original records. Data significantly deviating from clinically acceptable ranges must be verified, with necessary explanations provided by the form-filling physician/nurse and supervising physician.

**12. Quality Control and Quality Assurance**

12.1 Quality Control

1) Laboratory Quality Control Measures:

- Each participating hospital laboratory in the clinical trial should establish unified test indicators, standard operating procedures (SOPs), and quality control procedures.

2) Requirements for Investigators:

- Investigators should have the appropriate professional knowledge, qualifications, and research capabilities. Personnel should be relatively stable following qualification review.

3) Investigator Training:

- Investigators should fully understand and master the clinical trial protocol and the specific connotations of various indicators (e.g., study objectives, inclusion and exclusion criteria, prohibited medications). The specified objective indicators should be checked according to the planned time, place, and method.

- AEs or unexpected toxic side effects should be observed and followed up. The description of subjective symptoms should be objective, without leading or prompting.

- CRFs should be filled out promptly, truthfully, and accurately to ensure all conclusions of the clinical trial are based on original data.

- Blood specimens should be preserved and transported according to SOPs.

4) Measures to Ensure Subject Compliance:

- Participating physicians should patiently explain the trial to subjects to ensure they fully understand and cooperate with the trial.

5) Other Measures:

- Inform subjects about possible adverse reactions to the trial drugs. If any adverse reactions occur, they should immediately contact the physician or hospital.

12.2 Quality Assurance

- To ensure the quality of the trial, the investigator will develop a clinical research plan before the official start of the trial. The investigator must manage the study drug according to SOPs, including receiving, storing, distributing, and recalling. Study drugs must not be used for non-participants in this clinical trial, and must be ensured to be used only for the participants of this clinical trial.

- The Coordination Committee, consisting of all investigators, is responsible for the implementation of the entire trial, studying, and solving issues related to the trial.

- The investigator will appoint a monitor for this trial to ensure that the rights of the subjects are protected, the data recorded and reported in the trial are accurate, truthful, and complete, and that the trial follows the approved protocol and the GCP regulations.

12.3 Study Drug Management

- The sponsor is responsible for appropriately packaging and labeling the drugs used in the clinical trial, indicating that they are for clinical trial use only, and for transportation and handover. The clinical trial institution will designate personnel responsible for receiving and storing the drugs. The investigator is responsible for the use of the study drugs, ensuring that all study drugs are used only for the participants of the clinical trial. Dosage and usage should follow the trial protocol. The investigator will collect the study drugs on the day of the trial, with detailed records, including the investigator's signature and date.

- If the investigator receives or returns drugs due to changes in the number of participants, this must be recorded in detail.

- The records of drug use should be consistent with the actual amount used in the trial. All discrepancies must be verified or explained. After the trial, the remaining study drugs should be returned to the sponsor, with records of the returned quantity and the usage of the study drugs, signed and dated.

**13. Data Preservation and Summarization**

- The investigator must maintain original data for each patient (usually in the patient's medical record), from which the information on the CRF can be found, including a copy of the informed consent form signed by the patient with the trial number, containing the trial name, laboratory data, electrocardiograms, etc.

- The clinical trial responsibility unit should preserve the basic information of trial patients for a long time (usually five years after the end of the trial) according to national regulations. At that time, the investigator/institution will no longer retain records related to this trial. Basic data includes:

a). Approval from the Ethics Committee for the trial protocol and all protocol amendments.

b). All original data.

c). Case Report Forms (CRFs).

d). Informed consent forms.

e). Any other trial-related documents.

**14. Responsibilities of Parties and Publication of Papers**

- With the consent of the main project leader, researchers may publish papers and participate in academic conferences at home and abroad.

- When publishing papers, the research and development unit, production unit, and leading clinical research unit of the trial drug should be clearly indicated in the appropriate section of the paper.

- Rewards for clinical research papers belong to the clinical trial unit. When applying for medical achievement awards related to treatment methods or treatment technology, the clinical trial unit should consult with the drug development unit, production unit, and leading clinical research unit for joint application.

**15. Progress of the Trial**

- Enrollment start date: 12 January 2015

- Enrollment end date: 12 February 2022

- No. of enrolled patients: 51 cases

- Last follow-up date: 31 March 2023

**16. References**

1 Zhou, Y. *et al.* Global, regional, and national burden of acute myeloid leukemia, 1990-2021: a systematic analysis for the global burden of disease study 2021. *Biomark Res* **12**, 101 (2024).

2 Newell, L. F. & Cook, R. J. Advances in acute myeloid leukemia. *BMJ* **375**, n2026 (2021).

3 Dohner, H., Weisdorf, D. J. & Bloomfield, C. D. Acute Myeloid Leukemia. *N Engl J Med* **373**, 1136-1152 (2015).

4 DeWolf, S. & Tallman, M. S. How I treat relapsed or refractory AML. *Blood* **136**, 1023-1032 (2020).

5 Schlenk, R. F. *et al.* Impact of pretreatment characteristics and salvage strategy on outcome in patients with relapsed acute myeloid leukemia. *Leukemia* **31**, 1217-1220 (2017).

6 Ossenkoppele, G. & Lowenberg, B. How I treat the older patient with acute myeloid leukemia. *Blood* **125**, 767-774 (2015).

7 Senapati, J. *et al.* Lower intensity therapy with cladribine/low dose cytarabine/venetoclax in older patients with acute myeloid leukemia compares favorably with intensive chemotherapy among patients undergoing allogeneic stem cell transplantation. *Cancer* **130**, 3333-3343 (2024).

8 Stone, R. M. *et al.* Postremission therapy in older patients with de novo acute myeloid leukemia: a randomized trial comparing mitoxantrone and intermediate-dose cytarabine with standard-dose cytarabine. *Blood* **98**, 548-553 (2001).

9 Magina, K. N. *et al.* Cytarabine dose in the consolidation treatment of AML: a systematic review and meta-analysis. *Blood* **130**, 946-948 (2017).

10 Lowenberg, B. Sense and nonsense of high-dose cytarabine for acute myeloid leukemia. *Blood* **121**, 26-28 (2013).

11 Bae, S. H. *et al.* Bioassay for monitoring the anti-aging effect of cord blood treatment. *Theranostics* **9**, 1-10 (2019).

12 Lei, Q. *et al.* Extracellular vesicles deposit PCNA to rejuvenate aged bone marrow-derived mesenchymal stem cells and slow age-related degeneration. *Sci Transl Med* **13** (2021).

13 Sullivan, M. J. Banking on cord blood stem cells. *Nat Rev Cancer* **8**, 555-563 (2008).

14 Yun, H. D., Varma, A., Hussain, M. J., Nathan, S. & Brunstein, C. Clinical Relevance of Immunobiology in Umbilical Cord Blood Transplantation. *J Clin Med* **8** (2019).

15 Doessegger, L. & Banholzer, M. L. Clinical development methodology for infusion-related reactions with monoclonal antibodies. *Clin Transl Immunology* **4**, e39 (2015).
